# Eco-Friendly Multifunctional (TiO_2_/CoFe_2_O_4_)‑Biochar Nanocomposite for Arsenate Removal under Alkaline Conditions: Synthesis, Performance Evaluation, and Laboratory-Scale Assessment

**DOI:** 10.1021/acsomega.5c12881

**Published:** 2026-09-08

**Authors:** Maryam Shirinkar, João Carlos Alves Macedo, Claúdia Hitomi Watanabe, Elidiane Cipriano Rangel, André Henrique Rosa

**Affiliations:** Institute of Science and Technology, 28108 Sao Paulo State University—UNESP, Av Tres de março 511, Sorocaba, São Paulo 18087-315, Brazil

## Abstract

Titanium dioxide–cobalt ferrite functionalized sugarcane bagasse biochar (SBC/TiO_2_–CoFe_2_O_4_) was developed as a multifunctional adsorbent for arsenate [As­(V)] removal from water. While numerous binary TiO_2_-biochar or magnetic biochar systems have been reported, limited studies have explored the synergistic integration of both TiO_2_ and CoFe_2_O_4_ on a biochar matrix, particularly for arsenate removal under alkaline conditions. The material was synthesized via the sol–gel approach using sugarcane bagasse biochar pyrolyzed at 450 °C. Its adsorption performance toward arsenate was systematically evaluated at pH 8.5, selected due to its relevance to alkaline arsenic-contaminated groundwater and its proximity to the point of zero charge (PZC ≈7.8) of the composite. Surface and structural analyses using FTIR, SEM, and EDS provided evidence for the successful deposition of TiO_2_–CoFe_2_O_4_ particles onto the biochar matrix. Under the optimized adsorption conditions (0.1 g adsorbent, initial As­(V) concentration of 2 mg L^–1^), the composite achieved an experimental equilibrium adsorption capacity of 0.39 mg g^–1^, while 87.3% arsenic removal was obtained after 24 h of contact time. The adsorption kinetics followed the pseudo-second-order model. Nonlinear isotherm fitting indicated best overall agreement with the Redlich–Peterson model (*R* ^2^ = 0.669, AIC = −18.32), consistent with adsorption on an energetically heterogeneous composite surface; the Langmuir theoretical maximum capacity is reported as *q*_max_ = 0.526 mg g^–1^ for isotherm characterization purposes. Thermodynamic analysis confirmed a spontaneous (Δ*G*° < 0), endothermic (Δ*H*° > 0), entropy-driven adsorption process. ICP-OES analysis of the regenerant further demonstrated minimal Fe, Co, and Ti release (≤0.055% of the nominal metal content over four regeneration cycles), confirming high structural stability during repeated reuse. Overall, the SBC/TiO_2_–CoFe_2_O_4_ composite demonstrated promising multifunctionality by combining moderate adsorption capacity, magnetic recoverability, and reusability under environmentally relevant dilute conditions rarely tested in prior composite-biochar studies, highlighting its laboratory-scale potential as a sustainable biochar-based system for arsenate remediation in alkaline aqueous environments. Although phosphate competition experiments confirmed the expected inhibitory effect of competing oxyanions, further evaluation under complex natural groundwater conditions is warranted prior to field application.

## Graphical abstract

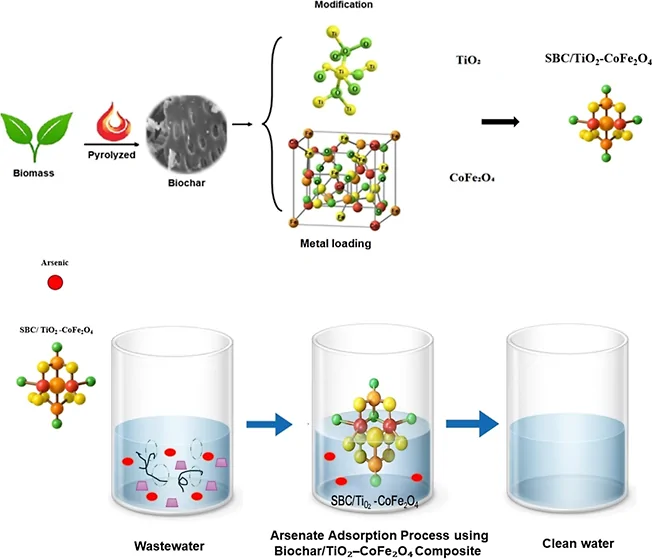

## 1 Introduction

Arsenic is a class I human carcinogen and one of the most hazardous inorganic contaminants in aquatic environments, causing severe toxic, mutagenic, and carcinogenic effects upon chronic exposure.^1^ It originates from natural geochemical processes including mineral weathering and sediment diagenesis, as well as anthropogenic sources such as mining, industrial effluents, and pesticide application.^2,3^ The World Health Organization (WHO) has set a maximum permissible limit of 0.01 mg L^–1^ (10 μg L^–1^) for arsenic in drinking water.^2^ Groundwater concentrations in affected regions of South Asia, Latin America, and sub-Saharan Africa frequently exceed this limit by one to 3 orders of magnitude, exposing an estimated 200 million people globally.^3,4^ In natural waters, arsenic occurs as arsenite [As­(III)] under reducing conditions and arsenate [As­(V)] under oxidizing conditions. As­(V) predominates in well-oxygenated (oxic) groundwater and is generally more amenable to adsorption-based removal.^5^ Competing anions such as phosphate, silicate, and sulfate further complicate remediation by competing for active surface sites.^9^

Several technologies have been applied for arsenic removal, including coagulation–flocculation, membrane filtration, ion exchange, electrocoagulation, oxidation–precipitation, and adsorption.^5–9^ Each carries significant operational and economic constraints. Coagulation–flocculation and electrocoagulation generate arsenic-rich sludge requiring regulated disposal, with reported treatment costs of 0.5–2.0 USD m^–3^.^6^ Membrane processes achieve high removal efficiencies but incur capital costs of 1–5 USD m^–3^ and are prone to fouling.^6,7^ Ion exchange resins are inhibited by these same competing anions and require costly regenerant disposal.^8^ Oxidation–precipitation demands prior conversion of As­(III) to As­(V), adding chemical dosing and process complexity.^9^ These constraints collectively highlight the critical need for low-cost, selective, and regenerable adsorbents suitable for decentralized water treatment.

Biochar, produced by thermochemical pyrolysis of biomass under oxygen-limited conditions, has attracted considerable attention as a low-cost adsorbent. It offers high specific surface area, porous architecture, and abundant surface functional groups including carboxyl, hydroxyl, and carbonyl moieties.^10,11^ Sugarcane bagasse, an abundant agricultural residue in Brazil, represents a renewable and low-cost biochar precursor that supports waste valorization and circular economy strategies.^11^ However, pristine biochar shows limited arsenate adsorption capacity and selectivity. Its predominantly negative surface charge at circumneutral pH repels arsenate anions (HAsO_4_^2–^), and it lacks the metal–oxygen–arsenic binding sites required for inner-sphere complexation.^10–12^

Surface modification with metal oxide nanomaterials has been widely explored to address these limitations. Titanium dioxide (TiO_2_) offers chemical stability, non-toxicity, and abundant surface hydroxyl groups (Ti–OH) that are hypothesized to facilitate arsenate complexation via ligand exchange at Ti^4+^ sites.^13,14,17,18^ Binary TiO_2_/biochar composites have shown markedly improved arsenate removal relative to unmodified biochar.^13,14,19^ Cobalt ferrite (CoFe_2_O_4_) nanoparticles provide a complementary strategy: they impart strong magnetic recoverability and mixed-valence Fe^3+^/Fe^2+^ active sites that facilitate arsenate binding through surface complexation.^12,15,16,20,21^ Magnetic biochar composites incorporating CoFe_2_O_4_ enable rapid adsorbent separation via external magnetic fields, addressing a key operational limitation of powdered adsorbents in field-scale systems.^15,16^ While Alomairy et al.^12^ previously reported this composite system for chlorophenol removal, its systematic application to arsenate adsorption under environmentally relevant dilute alkaline conditions has not been reported in studies identified through our literature search.

Despite these advances, three systematic gaps persist in the literature. First, binary biochar–iron oxide composites provide magnetic recoverability but lack the hydroxyl-rich TiO_2_ phase needed to increase hypothesized inner-sphere complexation site density.^22^ Second, TiO_2_/biochar systems achieve strong arsenate affinity but lack magnetic recoverability, limiting practical separation.^23^ Third, prior composite studies have predominantly evaluated performance at initial arsenic concentrations of 10–100 mg L^–1^, far exceeding the 0.5–5 mg L^–1^ range typical of contaminated groundwater, thereby overstating practical adsorption capacity.^24,25^ Furthermore, mechanistic characterization has generally been confined to single-framework kinetic or isotherm fitting, without multi-framework convergent validation integrating diffusion analysis, thermodynamic driving forces, and cross-model consistency. No studies identified in our literature search have simultaneously addressed all three gaps: magnetic recoverability, TiO_2_-mediated arsenate affinity, and evaluation under dilute groundwater-representative conditions for this composite system applied specifically to arsenate removal. Integrating TiO_2_ and CoFe_2_O_4_ within a single biochar matrix is therefore intended to simultaneously close the first two gaps identified above, combining inner-sphere complexation capacity with magnetic recoverability within one ternary platform while, the sugarcane bagasse-derived biochar provides structural support and supplementary adsorption domains.^26–28^ This ternary design is intended to combine, within a single material, functions that are individually but not jointly available in binary biochar–metal oxide systems, rather than to maximize adsorption capacity relative to either binary subsystem; it thereby targets the concurrent limitations of binary systems while enabling magnetic regeneration and operational simplicity.

Therefore, this study presents the synthesis and comprehensive characterization of a ternary SBC/TiO_2_–CoFe_2_O_4_ nanocomposite for arsenate removal under an environmentally relevant dilute conditions representative of contaminated groundwater (*C*_0_ = 2 mg L^–1^, pH 8.5). The adsorption behavior is investigated through pseudo-first- and pseudo-second-order kinetic modeling with multi-metric error analysis, four-model nonlinear isotherm characterization, Weber–Morris and Boyd diffusion analysis, van’t Hoff thermodynamic assessment, four-cycle magnetic regeneration evaluation, and laboratory-scale techno-economic analysis. To isolate and rigorously characterize the intrinsic adsorption mechanism, all experiments in this study were conducted using single-solute synthetic arsenate solutions under controlled laboratory conditions. The influence of the most critical competing groundwater anion, phosphate was evaluated through systematic competitive adsorption experiments reported in this study; the influence of silicate, sulfate, bicarbonate, and natural organic matter, together with validation of performance in real groundwater matrices, remains beyond the scope of this mechanistic-stage investigation and is identified as an essential next step prior to field-scale application.

This work aims to establish the mechanistic basis of arsenate uptake on this composite class and to define the operational boundaries for its laboratory-scale characterization; translational development toward practical groundwater arsenic remediation will require subsequent real-water matrix validation and pilot-scale evaluation.

## 2 Material and Methods

### 2.1 Materials

High purity chemicals (≥99% purity) involving: hydrochloric acid (HCl), titanium dioxide (TiO_2_), ethanol, citric acid, all purchased from Sigma-Aldrich, and cobalt­(II) nitrate hexahydrate, ferric nitrate nonahydrate, were purchased from Quimlab in São Paulo, Brazil. Sugarcane bagasse was sourced from Cafelandia city, São Paulo, Brazil. Double-distilled (DD) water was used to prepare all the synthesis solutions. Commercial, reagent-grade TiO_2_ was used rather than an in-house synthesized phase to ensure batch-to-batch consistency in purity, crystal structure, and particle size, thereby supporting reproducibility of this protocol by other laboratories. The anatase polymorph of TiO_2_ was confirmed by the supplier’s certificate of analysis (Sigma-Aldrich; ≥99% purity, anatase crystal form); use of a certified commercial phase eliminates batch-to-batch crystallographic variability and provides phase-identity assurance equivalent to that supplied by in-house XRD measurement for this specific component.

### 2.2 Production of Sugarcane–Based Biochar (SBC), CoFe_2_O_4_, SBC/TiO_2_–CoFe_2_O_4_ Composite

Pristine sugarcane bagasse biochar (SBC) was produced through thermal conversion in a muffle furnace operated in the limited presence of air. Approximately 10 g of dried sugarcane bagasse was loaded into the furnace per batch. Although an inert nitrogen atmosphere was considered, limited-air pyrolysis was selected because it is a simpler and more cost-effective method that, for cellulose- and hemicellulose-rich feedstocks such as sugarcane bagasse, is associated with a higher density of oxygen-containing surface functional groups (e.g., –COOH, –OH) relative to more strongly reducing (inert) pyrolysis environments.^29^ While limited-air pyrolysis may yield lower specific surface area than inert-atmosphere pyrolysis, the higher oxygenated functional group density achieved under these conditions was deliberately prioritized, as these groups serve as the primary anchoring sites for TiO_2_/CoFe_2_O_4_ deposition and arsenate coordination in this composite system. The temperature was raised at a constant rate of 5 °C min^–1^ until reaching 450 °C and maintained at this set point for 2 h to ensure complete carbonization. Afterward, the furnace was switched off and allowed to cool gradually to ambient temperature (25 ± 2 °C), yielding the as-produced biochar. The mass recovery efficiency of this pyrolysis step, calculated as the mass of biochar obtained relative to the initial mass of dried bagasse, was 20.0%. Cobalt ferrite (CoFe_2_O_4_) was prepared using a sol–gel method adapted from the procedure described by Alomairy et al.^12^ Cobalt­(II) nitrate hexahydrate (0.87 g, 0.1 M) and ferric nitrate nonahydrate (2.424 g, 0.2 M) were dissolved in 30 mL of deionized water and stirred magnetically at 600 rpm for 1 h at 25 ± 2 °C to obtain a uniform precursor solution, corresponding to the 1:2 (Co/Fe) stoichiometric ratio required by the CoFe_2_O_4_ spinel structure. This precursor preparation was scaled up proportionally across repeated batches to obtain sufficient CoFe_2_O_4_ powder for subsequent composite fabrication steps. In a separate step, 1.7292 g of citric acid was dissolved in 30 mL of deionized water and gradually added to the metal nitrate mixture. The combined solution was heated to 70 °C under continuous stirring until a homogeneous gel formed. Subsequently, the gel was dried at 120 °C for 12 h in a convection oven and then calcined in air at 600 °C for 2 h with a heating rate of 5 °C min^–1^, yielding CoFe_2_O_4_ powder. The mass recovery efficiency of this step, calculated relative to the combined mass of the metal nitrate and citric acid precursors used, was 42.8%.

The TiO_2_/CoFe_2_O_4_ composite was fabricated using a citrate-facilitated dispersion strategy, adapted from Alomairy et al.^12^ In the first step, 2 g of TiO_2_ was suspended in 60 mL of ethanol and stirred magnetically at 600 rpm for 1 h to promote uniform distribution. Citric acid (1.2 g) was then added to the TiO_2_/ethanol suspension and stirred for 60 min to chelate surface metal sites and promote uniform interparticle dispersion. Afterward, 2 g of previously synthesized CoFe_2_O_4_ was introduced into the mixture. The suspension was then subjected to centrifugation at 3500 rpm for 10 min to separate the solid phase. The collected material was rinsed twice with ultrapure water and dried at 70 °C for 3 h in a convection oven. Finally, thermal treatment was conducted in air at 600 °C for 2 h, at a heating rate of 5 °C min^–1^, yielding the TiO_2_/CoFe_2_O_4_ composite. The mass recovery efficiency of this step, relative to the combined mass of TiO_2_ and CoFe_2_O_4_ precursors (4 g), was 42.5%.

The preparation of the SBC/TiO_2_–CoFe_2_O_4_ was completed through an assembly procedure conducted under acidic adapted from Alomairy et al.^12^ First, 0.1 g of biochar was introduced into 10 mL of 0.5 M HCl. The mixture was stirred magnetically at 600 rpm for 1 h to promote activation of surface functional groups. Simultaneously, 0.9 g of TiO_2_/CoFe_2_O_4_ was dispersed in 50 mL of deionized water and stirred for 30 min to obtain a uniform suspension. Afterward, the two prepared dispersions were combined and exposed to ultrasonic irradiation at 30 kHz and 40 °C for 2 h to facilitate homogeneous incorporation of the components. The final suspension was separated by centrifugation and repeatedly rinsed with ultrapure water until a neutral pH was achieved. The collected solid was then dried in a hot-air oven at 100 °C for 24 h, yielding the SBC/TiO_2_–CoFe_2_O_4_ composite. The mass recovery efficiency of this final assembly step, relative to the combined mass of biochar and TiO_2_/CoFe_2_O_4_ inputs (1.0 g), was 78.0%.

### 2.3 Characterization of SBC, TiO_2_, CoFe_2_O_4_, and SBC/TiO_2_–CoFe_2_O_4_

The SBC, TiO_2_, CoFe_2_O_4_, and SBC/TiO_2_–CoFe_2_O_4_ were characterized using Fourier-transform infrared spectroscopy (FTIR; LUMOS II, Bruker) and energy-dispersive X-ray spectroscopy (EDS) coupled with scanning electron microscopy (SEM; JSM-6010LA).^4,12,30^

### 2.4 Batch Adsorption Experiments

To identify the optimal pH for arsenate adsorption and to characterize the surface charge behavior of the SBC/TiO_2_–CoFe_2_O_4_ composite, a preliminary pH study was conducted over the range pH 3–10 prior to the main adsorption experiments. The point of zero charge (PZC) of the composite was determined using the pH drift method (PZC = 7.8 ± 0.2). Batch experiments at each pH value were performed in duplicate (*n* = 2) at a fixed initial As­(V) concentration of 2 mg L^–1^, adsorbent dosage of 5.0 g L^–1^, and contact time of 24 h. Full results of the pH study, including removal efficiency at each pH value (±SD) and the PZC determination plot, are provided in the Supporting Information (Table S1 and Figures S1 and S2). Based on these results, pH 8.5 was selected for all subsequent main adsorption experiments because (i) it falls within the optimal removal window across the groundwater-relevant pH range (pH 6–9); (ii) it closely approximates the PZC of the composite (7.8 ± 0.2), hypothesized to favor chemisorptive uptake; and (iii) it represents the characteristic pH of arsenic-contaminated groundwater in the primary target application domain.^25^

All batch adsorption experiments were conducted at pH 8.5 with an initial As­(V) concentration of 2 mg L^–1^, employing a fixed adsorbent dosage of 5.0 g L^–1^ (0.1 g adsorbent in 20 mL solution) in 50 mL Falcon tubes. The As­(V) solution was adjusted to pH 8.5 using NaOH and HCl. Samples were placed on a mechanical shaker at 120 rpm at ambient temperature (25 ± 2 °C) and 2 mL aliquots were withdrawn at 0.5, 1, 4, 8, and 24 h at ambient temperature (25 ± 2 °C). Following filtration through a 0.45 μm PES membrane syringe filter, the filtrate was further centrifuged at 1000 g for 10 min prior to ICP-OES analysis, as an additional clarification step to minimize the potential carryover of fine suspended particles into the analyzed supernatant. All experiments were conducted in duplicate (*n* = 2) and results are reported as mean ± standard deviation (SD). The relative standard deviation (RSD) between duplicate measurements was below 1% for kinetic measurements, confirming excellent analytical reproducibility. The effect of initial concentration was evaluated over the range 0.5–6 mg L^–1^ (0.5, 1, 2, 4, and 6 mg L^–1^) with all other parameters held constant.

#### 2.4.1 Statistical Analysis

All experiments were conducted in duplicate (*n* = 2) and results are reported as mean ± SD. RSD values were below 1% for kinetic measurements and ranged from approximately 1–3.6% for equilibrium adsorption measurements, with isolated higher values at extreme pH conditions, indicating acceptable experimental reproducibility across all datasets. Triplicate repetition of isotherm and kinetic experiments is identified as a priority for follow-up work.

#### 2.4.2 Competitive Adsorption Experiments with Phosphate

To evaluate the effect of a competing anion on arsenate removal, batch competitive adsorption experiments were conducted with phosphate (PO_4_^3–^) as the primary interfering species. Phosphate was selected because of its structural analogy to arsenate; both are tetrahedral oxyanions, and its well-documented competition with arsenate for metal-oxide surface hydroxyl sites. Sodium phosphate dibasic (Na_2_HPO_4_) was used to prepare phosphate stock solutions at concentrations of 0, 2, 5, and 10 mg L^–1^ PO_4_^3–^. All competitive experiments were conducted under conditions identical to the main batch adsorption study: *C*_0_(As­(V)) = 2 mg L^–1^, pH 8.5, adsorbent dose = 5.0 g L^–1^ (0.1 g in 20 mL solution), contact time = 24 h, and temperature = 25 ± 2 °C. After the equilibration period, the adsorbent was separated magnetically, and the supernatant was analyzed for residual As­(V) by ICP-OES. Arsenate removal efficiency was calculated using eq 1. All experiments were performed in duplicate (*n* = 2) and results are reported as mean ± SD.

### 2.5 Removal Efficiency, Adsorption Kinetics, Isotherms, Thermodynamics, and Reusability

#### 2.5.1 Removal Efficiency and Adsorption Kinetics

The removal efficiency and adsorption capacities for the As­(V) were evaluated through eqs 1 and 2a,b), respectively.

1 $$Removal\;efficiency\;\left(\%\right)=\frac{C_{0}-C_{t}}{C_{0}}\times 100\%$$

2a $$Adsorption\;capacity\;\left(q_{t}\right)=\frac{\left(C_{o}-C_{t}\right)\times V}{W}$$

2b $$Adsorption\;capacity\;at\;equilibrium\;\left(q_{e}\right)=\frac{\left(C_{o}-C_{e}\right)\times V}{W}$$

where *C*_0_ (mg L^–1^) is the initial As­(V) concentration; *C*_*t*_ (mg L^–1^) is the As­(V) concentration at time *t*; *C*_e_ (mg L^–1^) is the As­(V) concentration at equilibrium (i.e., *C*_*t*_ at the final sampling time); *V* is the solution volume (0.02 L); and *W* is the adsorbent mass (0.1 g).

The adsorption kinetics were evaluated for the pseudo-first-order (PFO) kinetic model using (eq 3).^4^

3 $$\ln\left(q_{e}-q_{t}\right)=\left[\ln\left(q_{e}\right)-\left(k_{1}\right)\times t\right]$$

Where, *k*_1_ (h^–1^) is the pseudo-first-order rate constant; *t* is the contact time (h); *q*_(*t*)_ and *q*_e_ are the metal adsorption capacity at time = *t* and at equilibrium (mg g^–1^), respectively.

Data were evaluated for the pseudo-second-order (PSO) kinetic model using (eq 4).^31^

4 $$\frac{t}{q_{\left(t\right)}}=\frac{1}{{k_{2}q}_{e}^{2}}+\frac{t}{q_{e}}$$

Where *k*_2_ (g mg^–1^ h^–1^) is the pseudo-second-order rate constant; *t* is the contact time (h); *q*_(*t*)_ and *q*_e_ are the metal adsorption capacity at time = *t* and at equilibrium (mg g^–1^), respectively.

Adsorption kinetics and diffusion mechanisms were analysed using the Weber–Morris and Boyd models. The Weber–Morris intraparticle diffusion model eq 5a and the Boyd model eq 5b,c) to evaluate rate-limiting steps and distinguish between film diffusion and intraparticle diffusion

5a $$q_{t}=k_{id}\times t^{0.5}+C$$

where *q*_*t*_ (mg g^–1^) is the amount of adsorbate adsorbed at time *t*, *k*_id_ is the intraparticle diffusion rate constant, *C* (mg g^–1^) represents the boundary layer effect.

5b $$B_{t}=-0.4977-\ln\left(1-F\right)$$

5c $$F=\frac{q_{t}}{q_{e}}$$

where *F* is fractional attainment of equilibrium at time *t*, where *q*_*t*_ and *q*_e_ are the adsorbed amount at time *t* and at equilibrium, respectively. *B*_*t*_ is the Boyd constant related to the diffusion rate, used to distinguish film diffusion from intraparticle diffusion.

#### 2.5.2 Isotherm Models and Thermodynamics

The Langmuir isotherm assumes monolayer adsorption on a homogeneous surface with a finite number of identical binding sites. Langmuir isotherm fitting was performed, at concentrations 0.5, 1, 2, 4, and 6 mg L^–1^, using eq 6a,b.

6a $$Adsorption\;capacity\;\left(q_{e}\right)=\frac{q_{\max}K_{L}C_{e}}{1+K_{L}C_{e}}$$

The linearized form (eq 6b) is provided for reference only; all parameter estimation was performed by nonlinear regression

6b $$\frac{C_{e}}{q_{e}}=\frac{1}{K_{L}q_{\max}}+\frac{C_{e}}{q_{\max}}$$

Where *C*_e_ is the equilibrium concentration of the metal (mg L^–1^), *q*_max_ is the maximum amount of metal adsorbed (mg g^–1^), while *K*_L_ (L mg^–1^) is the Langmuir adsorption constant of metal affinity to the composite (adsorbent).

The Freundlich isotherm describes adsorption on heterogeneous surfaces. Freundlich isotherm fitting was performed using (eq 7).

7 $${\log q}_{e}=\log K_{F}+\frac{1}{n}\log C_{e}$$

where *n* and *K*_F_ ((mg g^–1^)­(L mg^–1^)^1/*n*^ are the Freundlich isotherm constants related to adsorption intensity and capacity.

The Temkin isotherm accounts for adsorbate–adsorbent interactions and is expressed by (eq 8).

8 $$q_{e}=B\ln\left(AC_{e}\right)$$

$$B=\frac{RT}{b},then\;q_{e}=\frac{RT}{b}\ln\left(AC_{e}\right)$$

where *q*_e_ (mg g^–1^) is the adsorption capacity at equilibrium, *C*_e_ (mg L^–1^) is the equilibrium concentration, *A* is the Temkin equilibrium binding constant (L mg^–1^), *b* (J mol^–1^) is the Temkin constant related to the heat of adsorption, and *B* = $\frac{RT}{b}$ (mg g^–1^) is a constant related to the heat of adsorption. *R* is the universal gas constant (8.314 J mol^–1^ K^–1^) and *T* is the experimental temperature (K).

The Redlich–Peterson isotherm was used to describe adsorption with a general exponential model, expressed by (eq 9)­

9 $$q_{e}=\frac{C_{e}K_{R}}{1+a_{R}C_{e}^{\beta }}$$

where *q*_e_ (mg g^–1^) is the equilibrium adsorption capacity, *C*_e_ (mg L^–1^) is the equilibrium concentration, *K*_R_ is the Redlich–Peterson equilibrium constant (L g^–1^), and *a*_R_ (L mg^–1^) is the heterogeneity factor indicating surface site distribution, and β (dimensionless, 0 < β ≤ 1) is the heterogeneity exponent, with β → 1 indicating Langmuir-like (homogeneous) behavior and β → 0 indicating Freundlich-like (heterogeneous) behavior.

A thermodynamic study was performed at 298, 308, and 318 K. The thermodynamic parameters were obtained from (eqs 10 and 11).^32–35^

10 $${\ln K}_{d}=\frac{\Delta S^{{}^{\circ}}}{R}-\frac{\Delta H^{{}^{\circ}}}{RT}$$

11 $$\Delta G^{{}^{\circ}}=\Delta H^{{}^{\circ}}-T\Delta S^{{}^{\circ}}$$

where *T* (K) is the absolute temperature; Δ*G*° (kJ mol^–1^) is the Gibbs free-energy change; Δ*H*° (kJ mol^–1^) is the enthalpy change; Δ*S*° (J mol^–1^ K^–1^) is the entropy change; and *R* is the universal gas constant (8.314 J mol^–1^ K^–1^).^36^

### 2.6 Reusability and Stability of the Adsorbent

The reusability of SBC/TiO_2_–CoFe_2_O_4_ was evaluated over four consecutive adsorption–desorption cycles under optimal conditions (adsorbent dose: 0.1 g; initial As­(V) concentration: 2 mg L^–1^; pH 8.5; temperature: 25 ± 2 °C; contact time: 24 h). After each adsorption cycle, the adsorbent was magnetically separated from solution and regenerated by rinsing with 20 mL of 5% HCl (v/v), followed by repeated washing with deionized water until neutral pH was achieved, and oven drying prior to reuse. The residual As­(V) concentration in the supernatant after each adsorption step was determined by ICP-OES to calculate the cycle-specific arsenate removal efficiency using eq 1.^4^

To evaluate the chemical stability of the composite during repeated regeneration, the spent 5% HCl regenerant solution collected after each adsorption–desorption cycle was separated from the adsorbent by centrifugation and subsequently analyzed by ICP-OES for Fe, Co, and Ti content. Results are reported in Supporting Information Table S9.

## 3 Results and Discussion

### 3.1 Molecular Structure, Elemental Composition, and Morphology of SBC, TiO_2_, CoFe_2_O_4_, and SBC/TiO_2_–CoFe_2_O_4_

#### 3.1.1 FTIR Analysis of SBC, TiO_2_, CoFe_2_O_4_, and SBC/TiO_2_–CoFe_2_O_4_ before and after Arsenate Adsorption

The Fourier-transform infrared (FTIR) spectra of SBC and the synthesized SBC/TiO_2_–CoFe_2_O_4_ composite (Figure 1a) provide qualitative insight into the surface functional groups and structural modifications associated with composite formation before arsenate adsorption at pH 8.5. The observed peak shifts and intensity variations suggest possible interactions between the biochar matrix and TiO_2_–CoFe_2_O_4_ components. The pure SBC exhibited characteristic absorption bands at 3443, 2336, 1706, 1615, 1209, 1093, and 930 cm^–1^, corresponding to the stretching vibrations of O–H, O–C–O, C–O, aromatic C–C, C–O, and C–O–C groups. The broad, intense band at 3443 cm^–1^ is attributed to the hydroxyl stretching of surface –OH groups and adsorbed water molecules.^37^ The weak absorption band near 2336 cm^–1^ reflects the asymmetric stretching vibration of CO_2_, typically attributed to physically adsorbed atmospheric CO_2_ or carbonate species (−CO_3_^2–^) weakly bound to the biochar surface.^4^ The band at 1706 cm^–1^ is attributed to C–O stretching vibrations of carboxylic functional groups. The feature at 1615 cm^–1^ arises from stretching of aromatic C–C or asymmetric stretching of carboxylate (COO^–^) groups and reflects the semi-graphitic character of the biochar matrix.^38^ The peaks observed at 1209 and 1093 cm^–1^ correspond to C–O stretching vibrations of phenolic, ether, and alcoholic components.^39^ The minor peak at 930 cm^–1^ represents C–H out-of-plane bending of aromatic C–H or C–O deformation vibrations. Collectively, these oxygenated functionalities constitute the principal active sites available for surface reactions and adsorption. The carbon matrix structure remained largely intact following functionalization with TiO_2_ and CoFe_2_O_4_, as the composite’s FTIR spectrum confirmed that the primary SBC bands were still detected. However, changes in peak intensity and slight shifts in a few peaks suggest that chemical bonding and interaction between the SBC and the metal oxides may have occurred. The O–H stretching band (3443 cm^–1^) was slightly diminished and shifted, indicating that hydroxyl groups were coordinated to Ti^4+^, Fe^3+^, and Co^2+^ during composite formation. The C–O and C–O stretching bands at 1706, 1209, and 1093 cm^–1^ exhibited a loss of intensity, supporting the view that oxygen-containing groups are essential for secure metal-oxide nanoparticle anchoring. Moreover, weak absorptions below 700 cm^–1^ can be attributed to Fe–O and Ti–O lattice vibrations, supporting the incorporation of TiO_2_ and CoFe_2_O_4_ phases.^40,41^ In summary, FTIR data indicate that oxygen-bridge bonds (C–O–M, where M = Ti, Fe, or Co) are responsible for the formation of the SBC/TiO_2_–CoFe_2_O_4_ composite, with surface –OH, C–O, and C–O groups serving as nucleation and coordination sites that promote structural integrity.

**Figure 1:**
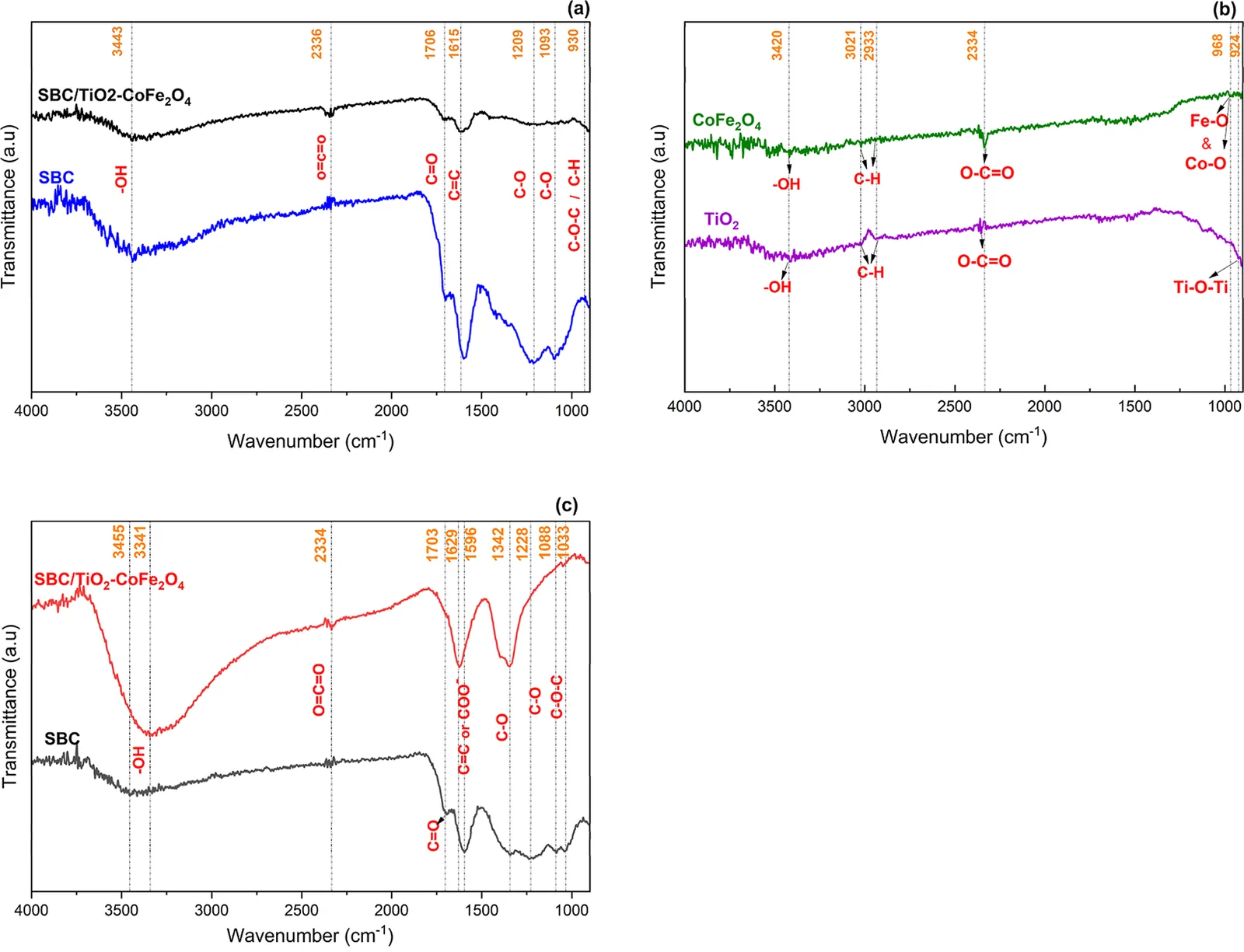
FTIR spectra of the (a) pre-adsorption of SBC and SBC/TiO_2_–CoFe_2_O_4_, (b) pre-adsorption of TiO_2_ and CoFe_2_O_4_, (c) post-adsorption of SBC and SBC/TiO_2_–CoFe_2_O_4_ at pH 8.5.

The main vibrational bands related to the structural and functional properties of TiO_2_ and CoFe_2_O_4_ are shown in Figure 1b. A broad absorption band appears at 3420 cm^–1^ for both materials, indicating stretching vibration of surface hydroxyl groups (−OH) and confirming the presence of surface hydroxyls or adsorbed water. This broad band is consistent with the inherent hydrophilicity of both materials, providing surface sites that are subsequently available for aqueous-phase interaction during adsorption.^42^ The peaks at 3021 and 2933 cm^–1^ are assigned to the asymmetric and symmetric C–H stretching vibrations, respectively, and reflect residual organic or weakly bound carbonaceous species on the surface of both TiO_2_ and CoFe_2_O_4_. The emergence of similar C–H vibrations in both spectra is consistent with residual carbonaceous contamination from the synthesis or calcination environment. The band near 2334 cm^–1^ is associated with stretching vibrations of atmospheric CO_2_ or surface-adsorbed carbonate species, typical for oxide-based materials exposed to air. In the low-frequency region, two prominent absorption bands at 968 and 924 cm^–1^, are consistent with metal–oxygen stretching vibrations: for TiO_2_, these bands represent Ti–O–Ti lattice vibrations; for CoFe_2_O_4_, they represent metal–oxygen (Fe–O and Co–O) bond vibrations within the spinel lattice structure.^43^ The overlap of these metal–oxygen bands indicates a common structural motif underlying the strong metal–oxygen bonding that contributes to the chemical stability and adsorption reactivity of both materials.^41^

The FTIR spectra of SBC and the synthesized SBC/TiO_2_–CoFe_2_O_4_ composite before and after arsenate adsorption (Figure 1c) provide qualitative insight into surface functional groups and possible interactions involved in the adsorption process. Variations in peak position and intensity suggest changes in surface chemistry following arsenate uptake. Following As­(V) adsorption, the single O–H band observed at 3443 cm^–1^ before adsorption broadened and resolved into two components at 3455 and 3341 cm^–1^, consistent with strengthened and more heterogeneous hydrogen bonding involving surface hydroxyl groups. While this change is compatible with hydroxyl-group participation in arsenate binding, the intensity and bandwidth changes alone cannot distinguish inner-sphere from outer-sphere coordination, and this assignment should therefore be regarded as indicative rather than confirmatory. The weak band attributed to the asymmetric stretching of O–C–O from surface carbonates (CO_3_^2–^) or adsorbed CO_2_ shifted only marginally from its pre-adsorption position (2336 → 2334 cm^–1^; Δ ≈ −2 cm^–1^, within instrumental resolution) and became more well-defined after sorption, possibly due to carbonation of metal sites modified by arsenate adsorption.^44^

The carboxylic C–O stretching band, observed at 1706 cm^–1^ before adsorption, shifted slightly to 1703 cm^–1^ (Δ ≈ −3 cm^–1^) and decreased in intensity after adsorption; it is therefore inferred that this functional group may participate in surface interaction with the arsenate ion, although FTIR alone cannot establish whether this interaction reflects direct coordination or a hydrogen-bonding-mediated effect. The aromatic C–C/asymmetric COO^–^ band, observed as a single feature at 1615 cm^–1^ before adsorption, resolved into two distinct components at 1629 and 1596 cm^–1^ after adsorption; this splitting indicates the emergence of two chemically distinct carboxylate/aromatic environments, possibly corresponding to unperturbed and arsenate-associated COO^–^ groups, and is consistent with possible ligand exchange or electrostatic interaction with arsenate.^45,46^ The peak at 1342 cm^–1^, corresponding to C–O stretching of carboxylates, also increased in intensity, suggesting increased involvement of this group in surface interaction, without indicating a specific binding mode.

The C–O and C–O–C stretching vibrations of phenolic, ether, and alcoholic groups, observed at 1209 and 1093 cm^–1^ before adsorption, shifted and resolved into three components at 1228, 1088, and 1033 cm^–1^ after adsorption. The phenolic C–O band displayed the largest displacement in this dataset, shifting from 1209 to 1228 cm^–1^ (Δ ≈ +19 cm^–1^), while the alcohol/ether C–O band at 1093 cm^–1^ resolved into two components at 1088 and 1033 cm^–1^ (Δ ≈ −5 and −60 cm^–1^, respectively). These changes indicate measurable involvement of phenolic and ether/alcohol oxygen donors as arsenate-binding sites.

Collectively, these spectral changes indicate that carboxylate, phenolic, and hydroxyl-bearing functional groups are measurably perturbed by arsenate uptake, consistent with surface-mediated chemisorption; FTIR data alone, however, cannot establish whether this perturbation reflects direct (inner-sphere) coordination or strong hydrogen-bonded (outer-sphere) association, a question further examined in the context of the elemental evidence below.

Figure 2 depicts the EDS elemental mapping and composition of pristine SBC and the SBC/TiO_2_–CoFe_2_O_4_ composite prior to adsorption at pH 8.5. Pristine SBC shows a carbon content of 85.2 wt % and an oxygen content of 14.6 wt %, indicative of its predominantly carbonaceous composition. For the composite, the carbon content is 19.2 wt %, accompanied by correspondingly higher amounts of oxygen (46.7%), titanium (29.7%), iron (2.7%), and cobalt (1.6%).

**Figure 2:**
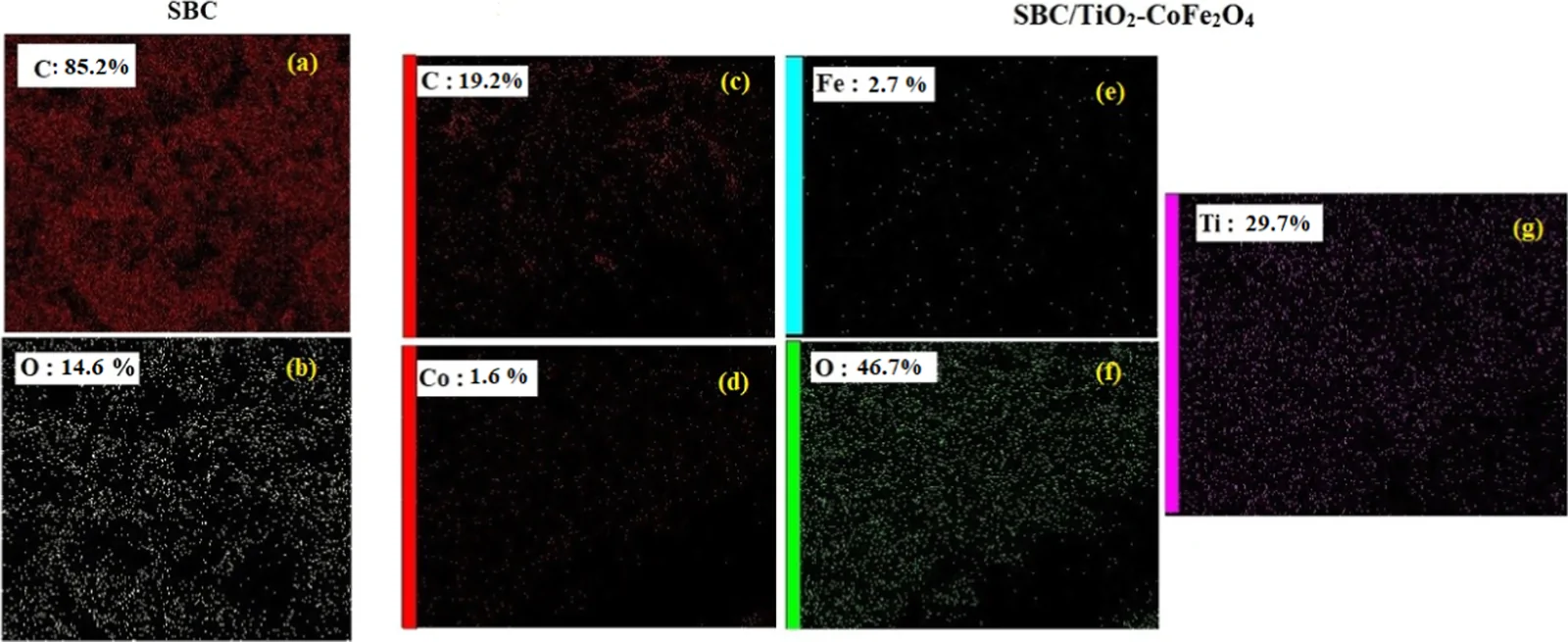
Pre-adsorption EDS elemental mapping and data (%) of composites of SBC (a,b) and SBC/TiO_2_–CoFe_2_O_4_ (c–g).

The EDS data shown in Figure 3 were collected following arsenate adsorption at pH 8.5. The pristine SBC material consisted predominantly of carbon (87.8 wt %) with a minor oxygen content (11.8 wt %). After adsorption, the results confirm successful arsenate uptake by the SBC/TiO_2_–CoFe_2_O_4_ composite, as evidenced by the clear presence of 2.8 wt % As­(V) in the elemental mapping (Figure 3h). In contrast, pristine SBC exhibited limited arsenic retention. For the composite relative to pristine SBC, the carbon content was lower (18.8 wt % vs 87.8 wt %) while the oxygen content was substantially higher (41.2 wt % vs 11.8 wt %), reflecting the contribution of the TiO_2_–CoFe_2_O_4_ oxide phases. In addition, titanium (31.4 wt %), cobalt (2.9 wt %), and iron (2.9 wt %) were clearly detected, confirming the successful incorporation of metal oxides into the SBC matrix. These results indicate that the introduction of Ti, Fe, and Co provided additional metal–oxygen active sites, which arsenate adsorption relative to pristine SBC.

**Figure 3:**
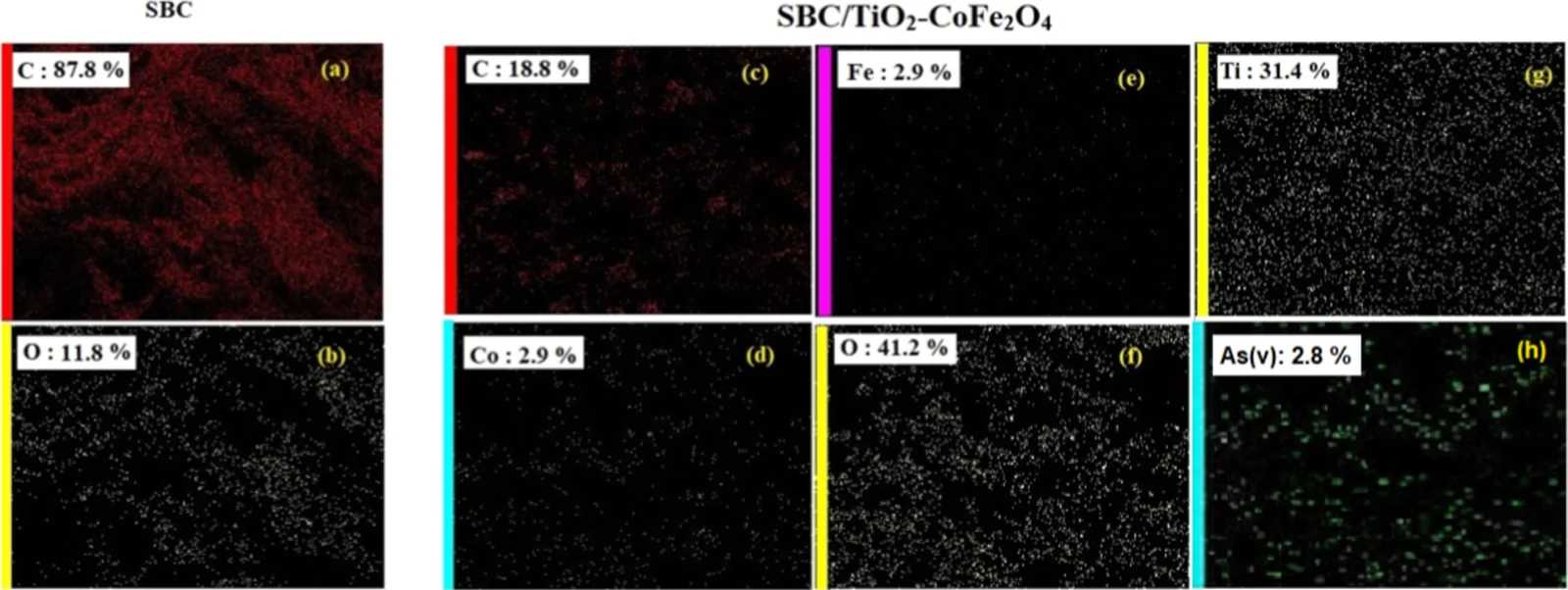
Post adsorption EDS elemental mapping and data (%) of composites of SBC (a,b) and SBC/TiO_2_–CoFe_2_O_4_ (c–h).

Comparing the composite’s elemental composition before and after adsorption (Figures 2 and 3), the oxygen content decreased slightly (46.7 → 41.2 wt %), while titanium, iron, and cobalt each showed modest relative increases (Ti: 29.7 → 31.4 wt %; Fe: 2.7 → 2.9 wt %; Co: 1.6 → 2.9 wt %). When these changes are summed-together with the newly appeared arsenic signal (+2.8 wt %) and the small decrease in carbon (19.2 → 18.8 wt %), the net change closely approaches zero (Δtotal ≈ +0.1 wt %). This near-complete mass-balance closure indicates that the redistribution among C, O, Ti, Fe, and Co is predominantly a renormalization effect arising from the appearance of the new arsenic signal, rather than independent evidence of selective elemental enrichment at arsenate-binding sites. Accordingly, the direct detection of arsenic itself, rather than secondary shifts in O, Ti, Fe, or Co content, constitutes the principal EDS-based evidence of arsenate uptake. This finding is compatible with ligand exchange between arsenate and surface hydroxyl functionalities (M–OH), hypothetically leading to inner-sphere M–O–As­(V) coordination; however, bulk elemental EDS data cannot, on their own, distinguish inner-sphere complexation from outer-sphere electrostatic association, since both binding modes would similarly increase the local arsenic signal.

It should also be noted that EDS interrogates a near-surface volume on the order of 1–2 μm, substantially larger than the sub-nanometer length scale over which surface complexation (inner- versus outer-sphere coordination) occurs. EDS can therefore confirm the presence, spatial distribution, and approximate quantity of adsorbed arsenic, but cannot resolve the local coordination environment or bonding geometry of the bound arsenate.

A comparative assessment of Figures 2 and 3 demonstrates a marked, arsenic-specific redistribution of elemental components within the composite following arsenic uptake, reflecting physicochemical interaction with arsenic species, whereas pristine SBC displayed only minor variation in its elemental profile, consistent with limited arsenic retention and reinforcing the conclusion that the incorporated metal oxides are primarily responsible for enhancing arsenate immobilization.

Taken together, the FTIR evidence of carboxylate, phenolic, and hydroxyl perturbation and the EDS-confirmed presence of arsenic at the composite surface are consistent with arsenate binding predominantly through ligand exchange and possible inner-sphere complexation at metal–hydroxyl (M–OH) and oxygen-donor organic sites, although neither technique alone confirms the specific coordination geometry (e.g., monodentate versus bidentate), which would require complementary methods such as XPS or zeta potential measurements.

SEM and EDS analyses provide complementary evidence of composite morphology and arsenate uptake. Figure 4a–c display the distinct morphologies of pristine SBC, TiO_2_, and CoFe_2_O_4_, respectively. The surface of SBC was porous with distinct cavities, while TiO_2_ and CoFe_2_O_4_ exhibited particulate and layered morphologies.

**Figure 4:**
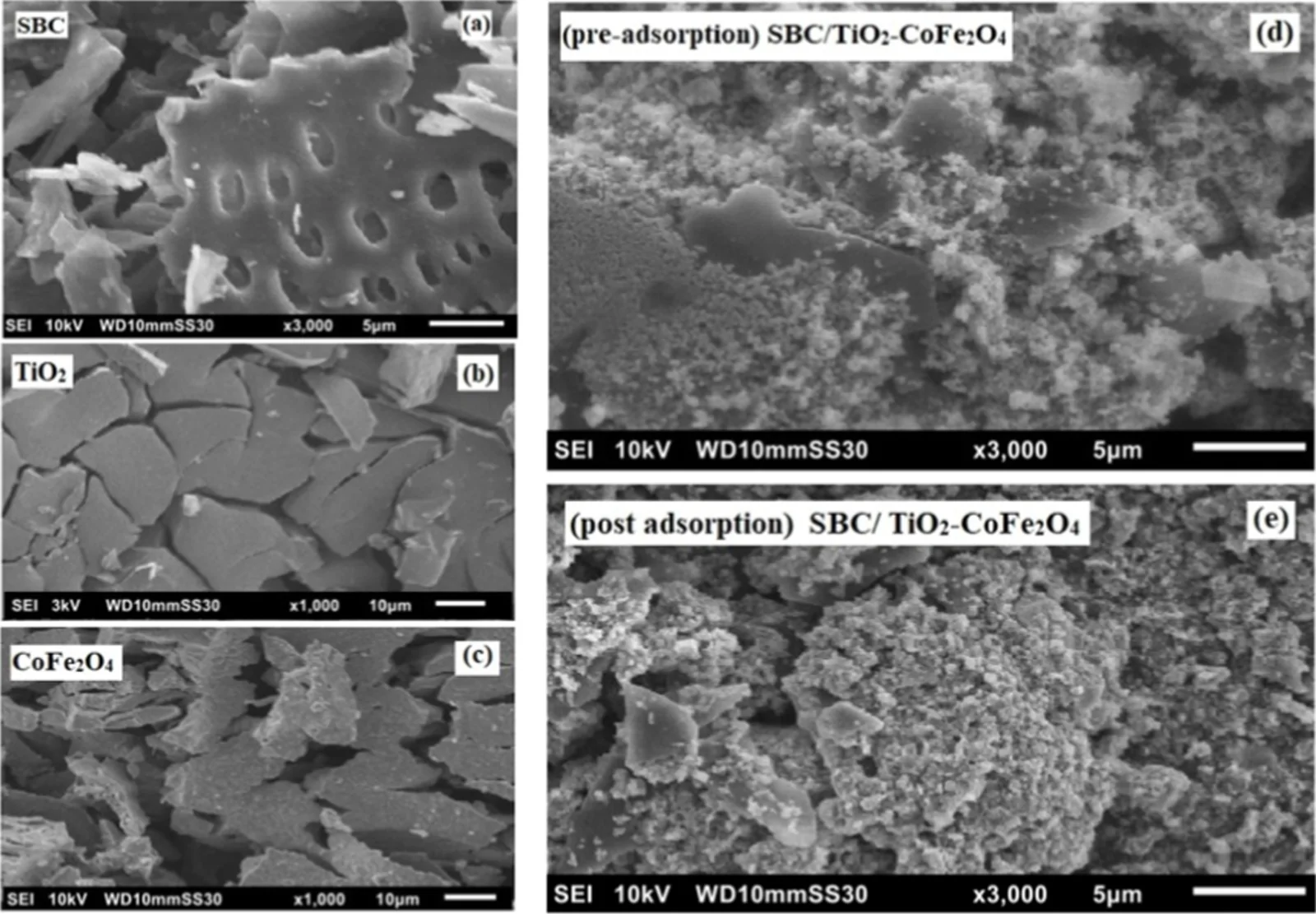
SEM micrographs of (a) SBC, (b) TiO_2_, (c) CoFe_2_O_4_, (d) SBC/TiO_2_–CoFe_2_O_4_ before arsenate adsorption, and (e) SBC/TiO_2_–CoFe_2_O_4_ after arsenate adsorption. The images reveal morphological changes associated with composite formation and arsenate uptake. SEM images were obtained at magnifications ranging from ×1000 to ×3000. Scale bars correspond to 5 μm for panels (a,d,e), and 10 μm for panels (b,c).

In the absence of arsenate, Figure 4d shows the surface morphology of SBC/TiO_2_–CoFe_2_O_4_ prior to adsorption. The composite surface exhibits rough and irregular morphology, with uniformly dispersed TiO_2_ and CoFe_2_O_4_ nanoparticles embedded within the biochar matrix. The increased surface roughness provides additional active sites and is expected to facilitate transport of adsorbate into the microporous biochar structure.

Following As­(V) adsorption, the composite surface (Figure 4e) exhibits a markedly altered morphology, with a denser appearance and partially filled pores. This reduction in porosity indicates that As­(V) species occupied and blocked available pore channels during adsorption. The observed transition from a porous to a more compact surface morphology is consistent with arsenate binding at surface hydroxyl groups associated with both Ti and Fe oxide domains, as supported by the EDS results.

Combined, these results suggest that arsenic was bound to the substrate through ligand exchange and, possibly, inner-sphere complexation with surface functional groups (M–OH).

The SEM and EDS analyses collectively indicate a synergistic effect of the composite’s components. SBC provides the backbone with an open and porous backbone, TiO_2_ provides surface hydroxyls for arsenate coordination, and CoFe_2_O_4_ not only contributes additional active sites but also enables magnetic recovery. Magnetic recoverability was confirmed operationally throughout all batch experiments: complete separation of the composite from solution was achieved within seconds using a hand-held NdFeB permanent magnet, without centrifugation or filtration. Quantitative VSM characterization (saturation magnetization, Ms) was not performed due to instrument availability constraints and is identified as a follow-up priority.

Taken together, the FTIR evidence of carboxylate, phenolic, and hydroxyl perturbation, the EDS-confirmed presence of arsenic at the composite surface, and the SEM-observed pore occlusion are consistent with arsenate binding to the composite predominantly through ligand exchange and possible inner-sphere complexation at metal–hydroxyl (M–OH) and oxygen-donor organic sites. It should be emphasized that none of these three techniques individually provides definitive proof of a specific coordination geometry; direct confirmation of inner- versus outer-sphere binding, or of monodentate versus bidentate coordination, would require complementary surface-sensitive techniques such as X-ray photoelectron spectroscopy (XPS), zeta potential measurements, or formal surface complexation modeling, which lie outside the scope of the present characterization.

### 3.2 Impact of Initial Concentration on As­(V) Removal

The adsorption experiments were conducted at a controlled pH of 8.5. This value was selected following a systematic pH-edge screening conducted across the range pH 3–10 (Supporting Information Figures S2 and S3, Table S1), which showed removal efficiency increasing from 44.2 ± 4.1% at pH 3 to a maximum of 99.4 ± 0.7% at pH 7, remaining above 98% through pH 8, and then declining to 82.9 ± 2.6% at pH 10. Across the pH 6–9 range typical of arsenic-contaminated groundwater, removal efficiency remained above 80% throughout, confirming that SBC/TiO_2_–CoFe_2_O_4_ maintains effective arsenate uptake across the full pH window relevant to most field deployment scenarios. Although pH 7 yielded the highest removal efficiency in this screening, pH 8.5 was deliberately selected for the detailed kinetic, isotherm, thermodynamic, and regeneration experiments because it represents a more environmentally realistic condition for alkaline arsenic-contaminated groundwater (typical range 6.5–8.5) and, by lying just above the composite’s point of zero charge (PZC ≈7.8 ± 0.2, Figure S2), allows the chemisorptive contribution to arsenate uptake to be evaluated under conditions where electrostatic attraction is reduced rather than dominant.^47–49^

At pH 8.5, the adsorbent surface is expected to carry a slight net negative charge, while arsenate predominantly exists as HAsO_4_^2–^ (p*K*_a_2__ = 6.94), with only a minor contribution from AsO_4_^3–^ (p*K*_a_3__ = 11.5). Although electrostatic repulsion between the negatively charged surface and arsenate anions would be expected to hinder adsorption, the relatively high removal efficiency observed (87.3 ± 0.1% at 2 mg L^–1^) suggests that specific surface interactions contribute significantly to arsenate uptake, likely involving ligand exchange at Lewis acid sites (Ti^4+^ and Fe^3+^/Co^2+^) that partially overcome unfavorable electrostatics. This mechanism has been reported for both TiO_2_ and spinel ferrites, where monodentate or bidentate surface complexes have been proposed even under mildly repulsive conditions (Section 3.1); however, the present spectroscopic and elemental data do not independently confirm this specific coordination geometry. The choice of pH 8.5 thus represents an appropriate compromise between mechanistic interpretability and environmental relevance, while avoiding excessive OH^–^ competition above pH 9–10.

Table 1 summarizes the influence of initial arsenate concentration (0.5, 1, 2, 4, and 6 mg L^–1^) on adsorption performance under fixed conditions (adsorbent dosage: 5.0 g L^–1^; contact time: 24 h; pH 8.5). Inter-replicate variation was low (SD ≤ 0.2%) across all concentration levels, indicating satisfactory reproducibility.

**Table 1:** Adsorption Efficiency & Adsorption Capacity of SBC/TiO_2_–CoFe_2_O_4_, with Dosage of 5.0 g L^–1^ (0.1 g Adsorbent in 20 mL Solution; pH 8.5; Contact Time: 24 h; *T* = 25 ± 2 °C; *n* = 2)^a^

*C*_0_ (mg As L^–1^)	removal efficiency (%) (mean ± SD)	RSD (%)	*q*_e_ (mg g^–1^)
0.5	82.4 ± 0.1	0.12	0.05
1	63.0 ± 0.1	0.16	0.13
2	87.3 ± 0.1	0.11	0.39
4	43.8 ± 0.2	0.46	0.36
6	45.8 ± 0.2	0.44	0.55

a *C*_0_: initial arsenic concentration; removal efficiency: percentage of As­(V) removed from solution, reported as mean ± SD of duplicate measurements (*n* = 2); SD: standard deviation; RSD: relative standard deviation, calculated as ( $\frac{SD}{mean}$ ) × 100%; RSD values for equilibrium adsorption capacity (*q*_e_) ranged from 1% to 3.6% as reported in Section 2.4.1 and Supporting Information Table S4. *q*_e_: equilibrium adsorption capacity (mg As removed per g adsorbent), calculated from mass balance.

The results in Table 1 show that arsenate uptake does not follow a simple monotonic trend with increasing initial concentration. At the lowest concentration tested (0.5 mg L^–1^), removal efficiency was relatively high (82.4 ± 0.1%) despite a low equilibrium capacity (*q*_e_ = 0.05 mg g^–1^), consistent with accessible binding sites substantially exceeding the available arsenate at this dilute condition. At 1 mg L^–1^, removal efficiency decreased to 63.0 ± 0.1% (*q*_e_ = 0.13 mg g^–1^), before recovering to a maximum of 87.3 ± 0.1% at 2 mg L^–1^, where *q*_e_ also reached 0.39 mg g^–1^. At 4 mg L^–1^, removal efficiency declined sharply to 43.8 ± 0.2%, and *q*_e_ itself decreased modestly to 0.36 mg g^–1^ relative to the 2 mg L^–1^ condition. The reproducibility of this decrease was confirmed by the duplicate measurements (RSD = 0.2% between replicates at *C*_0_ = 4 mg L^–1^; Supporting Information Table S4), ruling out random experimental error as its cause. Both removal efficiency and *q*_e_ increased again at 6 mg L^–1^, reaching 45.8 ± 0.2% and 0.55 mg g^–1^ (the highest value recorded), respectively.

This non-monotonic behavior in *q*_e_, rather than indicating experimental inconsistency, is consistent with progressive, non-uniform occupation of a heterogeneous composite surface comprising energetically distinct Fe–OH, Ti–OH, and biochar carbon-associated domains, as the relative contribution of high- and lower-affinity binding sites shifts with increasing initial concentration.

Overall, *q*_e_ increased more than 10-fold across the tested concentration range (0.05 to 0.55 mg g^–1^), demonstrating a clear concentration-dependent adsorption response.^47–49^

### 3.3 Adsorption Kinetics, Isotherm Models, and Thermodynamics

#### 3.3.1 Kinetic Analysis

The adsorption kinetics of arsenate onto SBC/TiO_2_–CoFe_2_O_4_, were evaluated using the pseudo-first-order (PFO) and pseudo-second-order (PSO) kinetic models (Figure 5). All kinetic experiments were conducted under conditions identical to those of the equilibrium studies (pH 8.5, adsorbent dosage = 5.0 g L^–1^, temperature = 25 ± 2 °C), ensuring direct comparability between kinetic and isotherm analyses.

**Figure 5:**
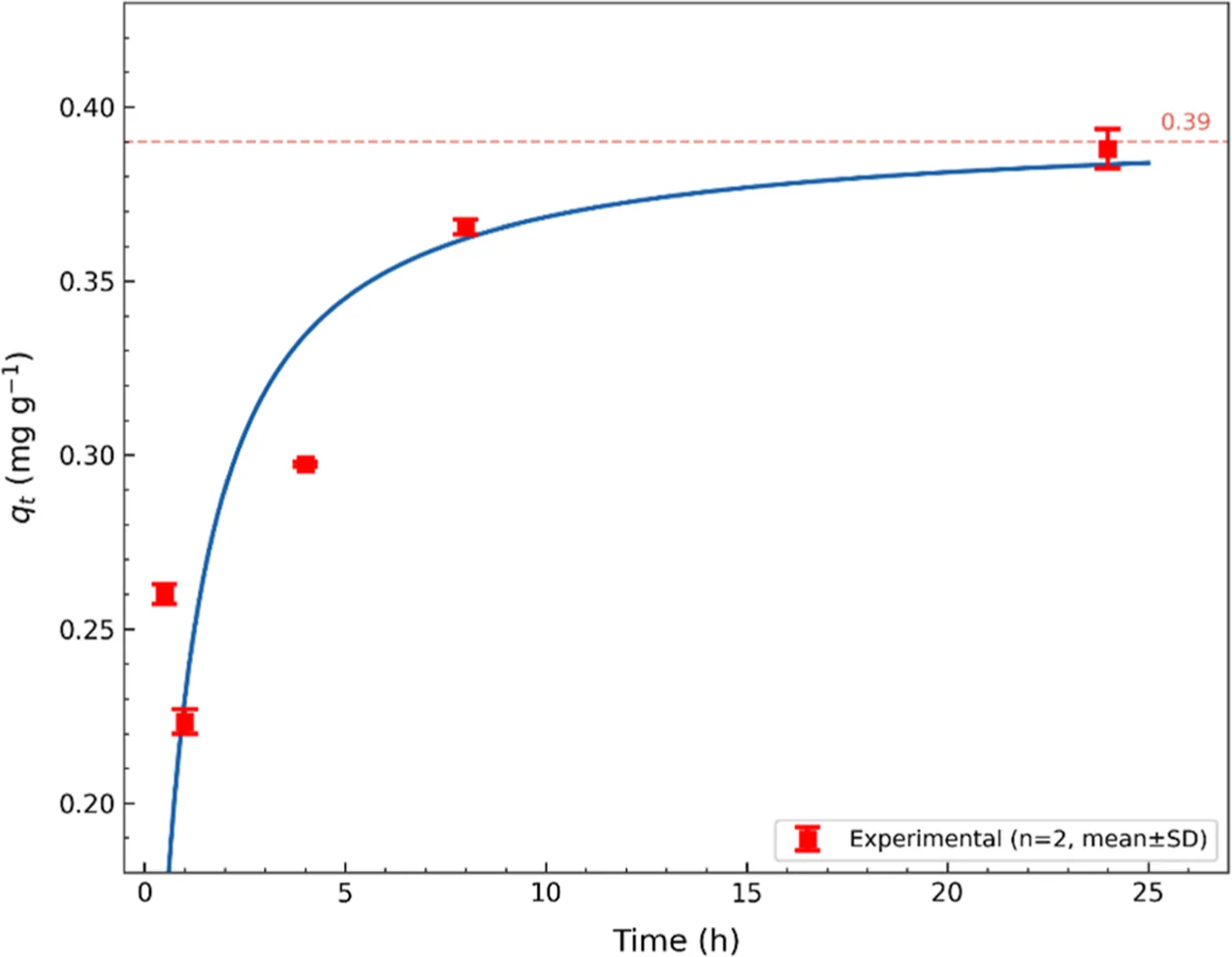
Adsorption kinetics of the composites SBC/TiO_2_–CoFe_2_O_4_ on As­(V) at pH 8.5. Data represent mean ± SD of duplicate experiments (*n* = 2, mean RSD = 0.99%). The dashed horizontal line indicates the experimentally reported equilibrium adsorption capacity (*q*_e_ = 0.39 mg g^–1^) used for kinetic model fitting.

To provide a comprehensive assessment of model performance, kinetic fitting was evaluated not only using correlation coefficients (*R* ^2^) but also through nonlinear error functions including chi-square (χ^2^), root mean square error (RMSE), and average relative error (ARE), as widely recommended for adsorption model evaluation.^50^

Model discrimination between PFO and PSO was based on the convergence of five independent criteria: the correlation coefficient (*R* ^2^), the three nonlinear error functions (χ^2^, RMSE, ARE), and the closeness of the model-predicted equilibrium capacity (*q*_e,cal_) to the experimentally measured value (*q*_e,exp_). A model was considered statistically preferred only when it outperformed the alternative model across all five criteria simultaneously, rather than on the basis of any single metric. Although the adsorption rate had substantially decreased by 24 h, a slow approach to equilibrium remained evident; accordingly, the reported *q*_e_ values represent conservative estimates of the true equilibrium capacity, and slightly higher uptake may be achieved with extended contact times.

The integrated interpretation of kinetic and equilibrium findings enabled further clarification of the adsorption pathway, which is conceptually depicted in Figure 6. The corresponding kinetic fitting results are displayed in Figure 7a,b. Furthermore, models grounded in diffusion theory were applied to examine the possible roles of mass transfer and internal diffusion processes. Specifically, the Weber–Morris intraparticle diffusion and Boyd models are presented in Figure 8a,b.

**Figure 6:**
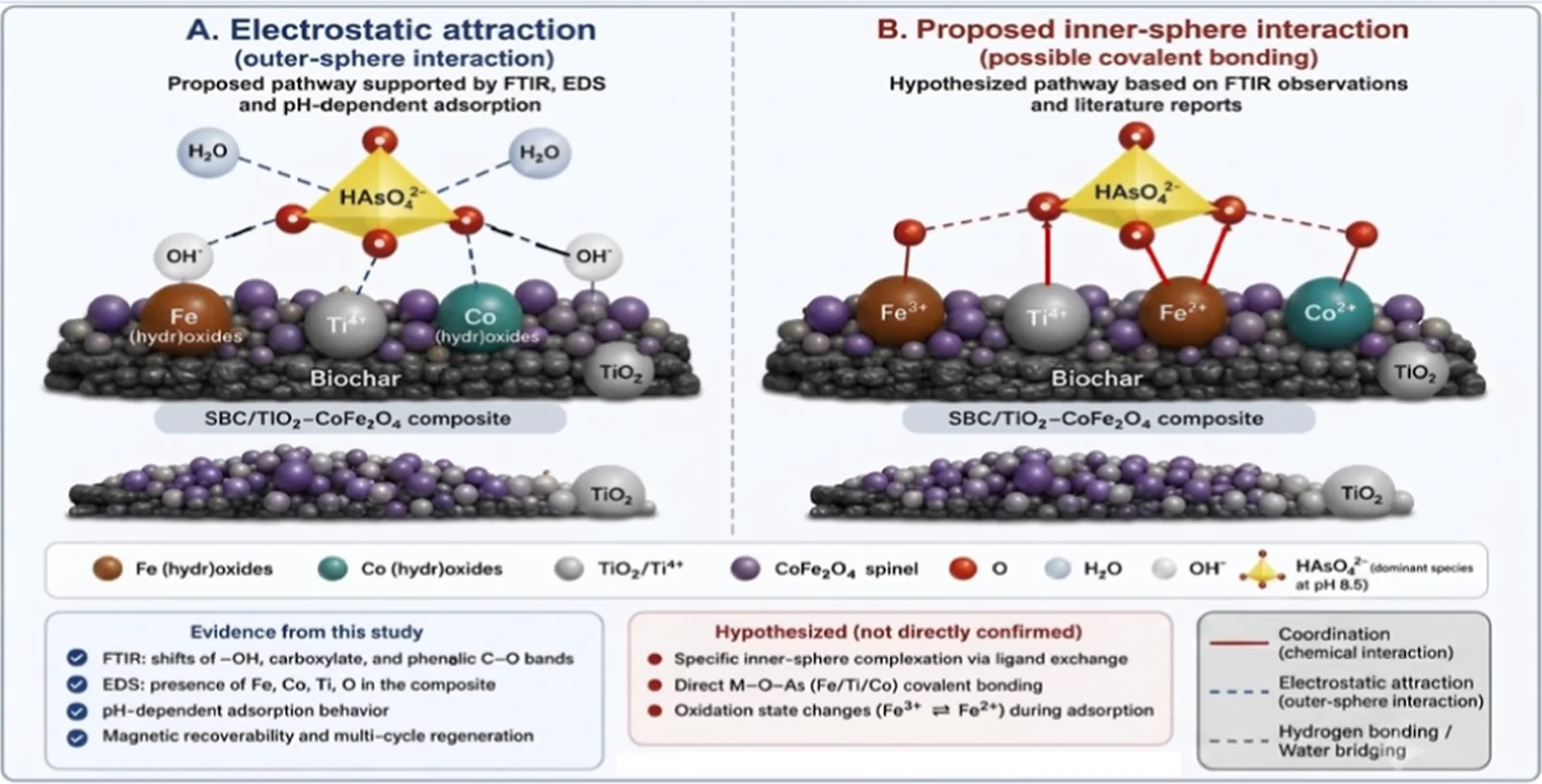
Proposed arsenate adsorption mechanisms onto the SBC/TiO_2_–CoFe_2_O_4_ composite. (A) Electrostatic attraction (outer-sphere interaction), consistent with FTIR, EDS, and pH-dependent adsorption results. (B) Proposed inner-sphere interaction (possible covalent bonding), hypothesized from FTIR band shifts and literature precedent; this binding mode has not been confirmed by XPS or EXAFS analysis and is presented as a working hypothesis only. Note: The visual layout and graphical elements of this figure were generated with the assistance of an AI-based illustration tool (graphical design only); the scientific content, mechanistic framework, and all interpretive assignments were exclusively conceived, developed, and validated by the authors. This figure represents a conceptual schematic of hypothesized adsorption pathways and does not imply confirmed coordination geometry or binding stoichiometry.

**Figure 7:**
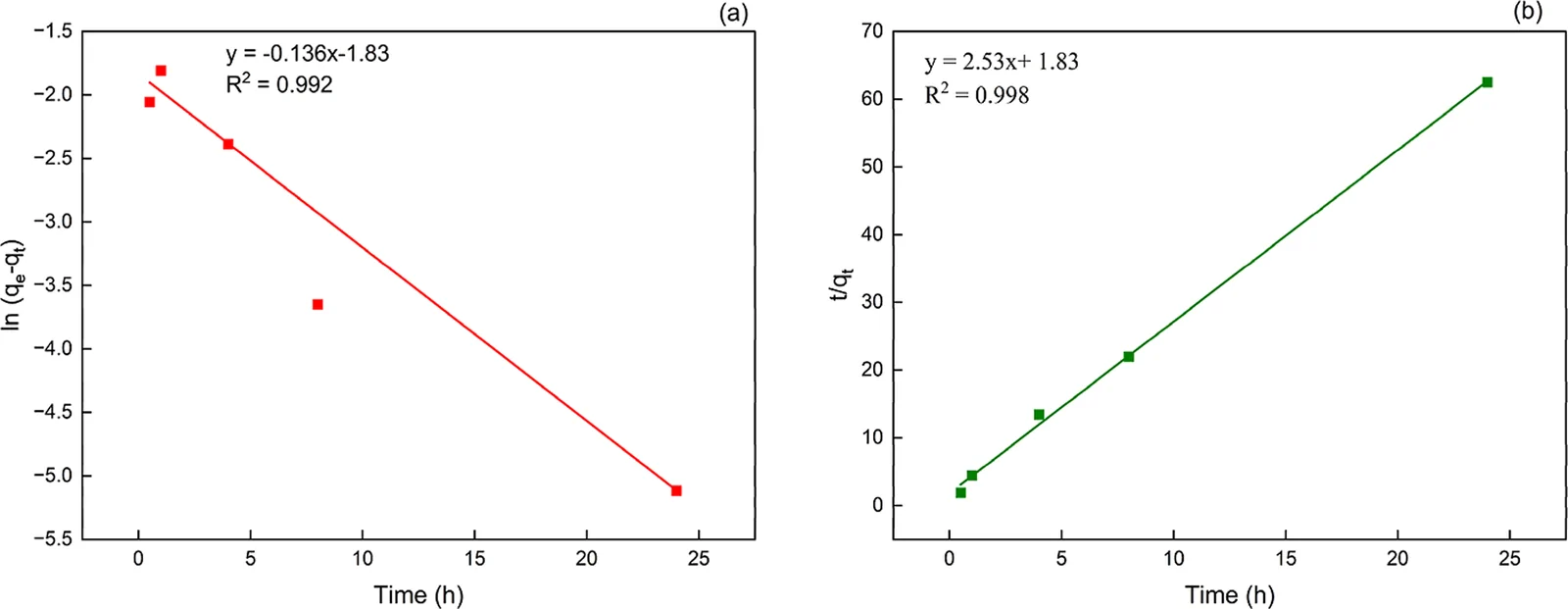
PFO (a) and PSO (b) model for SBC/TiO_2_–CoFe_2_O_4_ vs As­(V) at pH 8.5.

**Figure 8:**
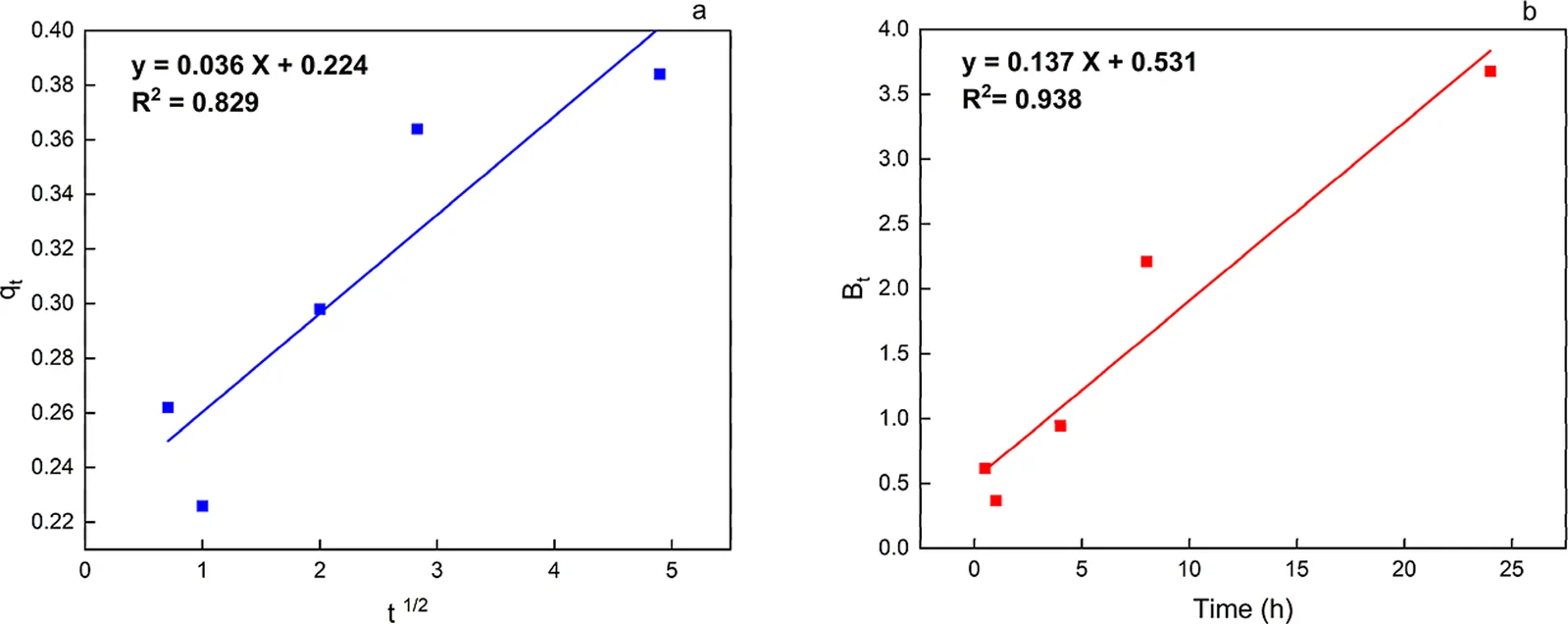
Weber–Morris intraparticle diffusion (a) and Boyd film diffusion (b) plots for arsenate adsorption onto the SBC/TiO_2_–CoFe_2_O_4_ composite at pH 8.5.

The adsorption of arsenate onto the SBC/TiO_2_–CoFe_2_O_4_ composite at pH 8.5 can be interpreted as a multistep process governed by the coupled effects of mass transfer, surface interactions, and site-specific binding under dilute and environmentally relevant conditions. Initially, arsenate transport from the bulk solution to the composite surface is driven by concentration gradients across the liquid boundary layer, followed by diffusion toward accessible adsorption sites.^51^

Given the porous architecture of the biochar matrix, intraparticle diffusion may contribute to arsenate migration toward internal reactive domains; however, the diffusion analyses indicate that arsenate uptake cannot be attributed to a single transport pathway and is more likely governed by the combined contribution of external mass transfer and surface interaction processes.^52,53^

Upon reaching the composite surface, arsenate adsorption proceeds through two coexisting interaction pathways: outer-sphere electrostatic association and hypothesized inner-sphere complexation at metal-oxide active sites (Figure 6). Outer-sphere interactions involve the electrostatic attraction of HAsO_4_^2–^ species (predominant at pH 8.5), to hydroxylated Fe- and Ti-containing surface domains, without the formation of direct metal–oxygen–arsenic bonds.^54,55^ These interactions are generally considered weak and reversible and may contribute primarily during the initial stages of adsorption.^54^

In parallel, inner-sphere complexation can occur through ligand exchange between arsenate oxyanions and surface hydroxyl groups on Fe- and Ti-containing active sites, resulting in the formation of Fe–O–As surface complexes.^54–56^ As illustrated schematically in Figure 6, both monodentate and bidentate coordination modes are plausible on the heterogeneous oxide surface.^55,56^

The TiO_2_ phase acts as a chemically stable, hydroxyl-rich support that improves nanoparticle dispersion and enhances the accessibility of reactive adsorption sites, thereby facilitating arsenate binding under mildly alkaline conditions.^57^

The kinetic data fit to the pseudo-first-order (PFO) model (Figure 7a) yielded a relatively high linearized correlation coefficient (*R* ^2^ = 0.992); however, this value alone is not a reliable basis for model selection, since linearization is known to distort the underlying error structure of kinetic data.^58^ Evaluated against the nonlinear error criteria, the PFO model performed markedly worse, yielding χ^2^ = 2.960, RMSE = 0.153 mg g^–1^, and ARE = 48.9%. Furthermore, the equilibrium adsorption capacity predicted by the PFO model (*q*_e,cal_ = 0.160 mg g^–1^) differed substantially (by 59%) from the experimentally determined value (*q*_e,exp_ = 0.39 mg g^–1^), indicating poor agreement between model predictions and observed adsorption behavior. It is noteworthy that high *R* ^2^ values obtained from linearized kinetic equations do not necessarily guarantee superior model performance, as mathematical linearization may alter the original error structure of adsorption data; therefore, model selection was based on the combined interpretation of *R* ^2^ values, adsorption-capacity agreement, and nonlinear error analyses rather than on correlation coefficients alone.^50^

The PSO model, by comparison provided consistently improved agreement with the experimental data. The PSO model exhibited a higher correlation coefficient (*R* ^2^ = 0.998), while the predicted equilibrium adsorption capacity (*q*_e,cal_ = 0.395 mg g^–1^) closely matched the experimentally determined value (*q*_e,exp_ = 0.39 mg g^–1^), corresponding to a deviation below 2%. Substantially lower error values were also obtained (χ^2^ = 0.067, RMSE = 0.048 mg g^–1^, and ARE = 10.5%), indicating superior predictive capability relative to the PFO model.

The 95% confidence interval for *q*_e_ (0.369–0.424 mg g^–1^) was correspondingly narrow, confirming precise estimation of this parameter. The confidence interval for *k*_2_ (1.13–10.83 g mg^–1^ h^–1^), in contrast, was considerably wider, reflecting the larger standard error and marginal statistical significance of the intercept term (*p* = 0.066). This wide interval represents a genuine limitation of the kinetic parameter estimation and indicates that the precise value of *k*_2_ should be interpreted with caution. Additional kinetic data points at shorter time intervals, particularly in the 0–60 min range where the adsorption rate is highest would be expected to substantially reduce this uncertainty by better constraining the initial curvature of the kinetic profile; this is identified as a recommended refinement for follow-up work. This wider interval for *k*_2_ does not, however, undermine the overall model selection, which remains supported by the convergence of *R* ^2^, χ^2^, RMSE, and ARE metrics favoring PSO over PFO.

Collectively, the PSO model outperformed the PFO model across every evaluation criterion simultaneously, *R* ^2^, χ^2^, RMSE, ARE, and *q*_e_ agreement (Table 2). This convergence of independent statistical evidence, indicates that the PSO model provides the most appropriate representation of arsenate adsorption kinetics among the models evaluated. The close agreement between calculated and experimental adsorption capacities is consistent with surface-mediated chemisorption as the dominant uptake mechanism for arsenate by the SBC/TiO_2_–CoFe_2_O_4_ composite.^58–60^

**Table 2:** Kinetic Parameters and Goodness-of-Fit Metrics for PFO and PSO Models Describing As­(V) Adsorption onto SBC/TiO_2_–CoFe_2_O_4_ (Initial As­(V) Concentration = 2 mg L^–1^, pH 8.5, *n* = 2, Mean ± SD)^a^

parameter	PFO	PSO
*q*_e_ cal (mg g^–1^)	0.160 (95% CI: 0.104–0.245)	0.395 (95% CI: 0.369–0.424)
*q*_e_ exp (mg g^–1^)	0.39	0.39
rate constant	*k*_1_ = 0.136 h^–1^ (95% CI: 0.102–0.172)	*k*_2_ = 3.498 g mg^–1^ h^–1^ (95% CI: 1.13–10.83)
*R* ^2^ (linearized)	0.992	0.998
χ^2^	2.9600	0.0668
RMSE (mg g^–1^)	0.1525	0.0479
ARE (%)	48.927	10.534

a χ^2^, RMSE, and ARE were calculated from nonlinear model predictions to provide comprehensive model evaluation beyond *R* ^2^.

Taken together, the kinetic and diffusion analyses support a multistep adsorption process involving external mass transfer followed by surface complexation at Fe- and Ti-containing active sites on the heterogeneous composite surface.^59^

#### 3.3.2 Diffusion-Based Kinetic Analysis

The Weber–Morris intraparticle diffusion model was applied to evaluate the extent to which internal diffusion influences arsenate adsorption onto SBC/TiO_2_–CoFe_2_O_4_ composite. The *q*_*t*_ versus *t* ^1/2^ plot (Figure 8a and Table 3) demonstrates an approximately linear relationship (*R* ^2^ = 0.8296). The intraparticle diffusion rate constant was determined as *k*_id_ = 0.036 mg g^–1^ h^–1/2^. However, because the extrapolated line does not pass through the origin and exhibits a positive intercept (*C* = 0.224 mg g^–1^), intraparticle diffusion is not the sole rate-controlling mechanism. The magnitude of this intercept reflects the boundary layer thickness and confirms that external mass transfer processes operate concurrently with intraparticle diffusion.

**Table 3:** Summary of Diffusion Kinetic Analysis

model	plot characteristics	key observation	interpretation
Weber–Morris	linear, does not pass through origin (*R* ^2^ = 0.8296)	intercept ≠ 0	intraparticle diffusion contributes but is not the sole rate-limiting step
Boyd	Linear, does not pass through origin (*R* ^2^ = 0.9384)	intercept ≠ 0	film diffusion dominates the adsorption rate
PFO/PSO	PSO provides better fit	higher *R* ^2^ and lower error	**chemisorption** significantly contributes to kinetics
overall mechanism	multistep process	—	**combined film diffusion**, **intraparticle diffusion, and surface reactions**

The Boyd model was then applied to differentiate whether film diffusion or intraparticle diffusion dominates the overall adsorption rate. As shown in the Boyd plot (Figure 8b and Table 3), a strong linear relationship was observed (*R* ^2^ = 0.9384); however, the extrapolated line yields a positive intercept of 0.531, indicating that the line does not pass through the origin and that film diffusion contributes more substantially than intraparticle diffusion to the rate-limiting process, with a partial intraparticle contribution also present. This finding suggests that a significant portion of the mass-transfer resistance resides in the liquid boundary layer surrounding the adsorbent particles. At pH 8.5, arsenate exists predominantly as HAsO_4_^2–^, a divalent anion whose electrostatic interaction with the composite surface requires overcoming a boundary layer energy barrier before surface binding can occur, a condition that favors a substantial film-diffusion contribution to the overall rate.

Taken together, the Weber–Morris (*k*_id_ = 0.036 mg g^–1^ h^–1/2^, *C* = 0.224 mg g^–1^) and Boyd (intercept = 0.531) analyses reveal a multistep adsorption process in which film diffusion governs the initial rate-limiting transport stage, while intraparticle diffusion into the porous biochar matrix contributes at later stages as external surface sites become progressively occupied. These diffusion findings are the superior performance of the PSO model (χ^2^ = 0.067, RMSE = 0.048 mg g^–1^, ARE = 10.5%), supporting the dominance of chemisorption-type surface interactions as the terminal binding step following mass transfer.

#### 3.3.3 Isotherm Models and Thermodynamics

The equilibrium adsorption behavior of arsenate onto the SBC/TiO_2_–CoFe_2_O_4_ composite at pH 8.5 was characterized using four isotherm frameworks of increasing mechanistic complexity: Langmuir, Freundlich, Temkin, and Redlich–Peterson. All parameters were determined by nonlinear regression, adopted in preference to linearized fitting because mathematical transformation of isotherm equations systematically distorts the error structure, introduces heteroscedasticity, and produces biased parameter estimates.^49,61^ The five equilibrium concentration points (*C*_e_ = 0.091–3.32 mg L^–1^) were selected to represent the Henry’s law region, transitional curvature, and near-saturation plateau, the three functional zones required for reliable isotherm parameter estimation, consistent with published practice for arsenate-composite systems.^22^ Model performance was evaluated using a comprehensive suite of error metrics (SSE, RMSE, MAE, χ^2^, AIC; Table 5), and residual analyses are provided in Supporting Information Figures S4 and S5. The nonlinear fitting results are presented in Figures 9a,b and 10a,b, and isotherm parameters are summarized in Tables 4 and 6. As noted in Section 3.2, the experimental *q*_e_ value at *C*_0_ = 4 mg L^–1^ was lower than at 2 mg L^–1^, precluding a strictly monotonic *q*_e_–*C*_e_ relationship as assumed by all four isotherm models; this non-monotonicity contributes directly to the moderate *R* ^2^ values obtained across all four models (0.611–0.673; Figure 9, Tables 4 and 5), consistent with genuine surface heterogeneity of the composite.

**Figure 9:**
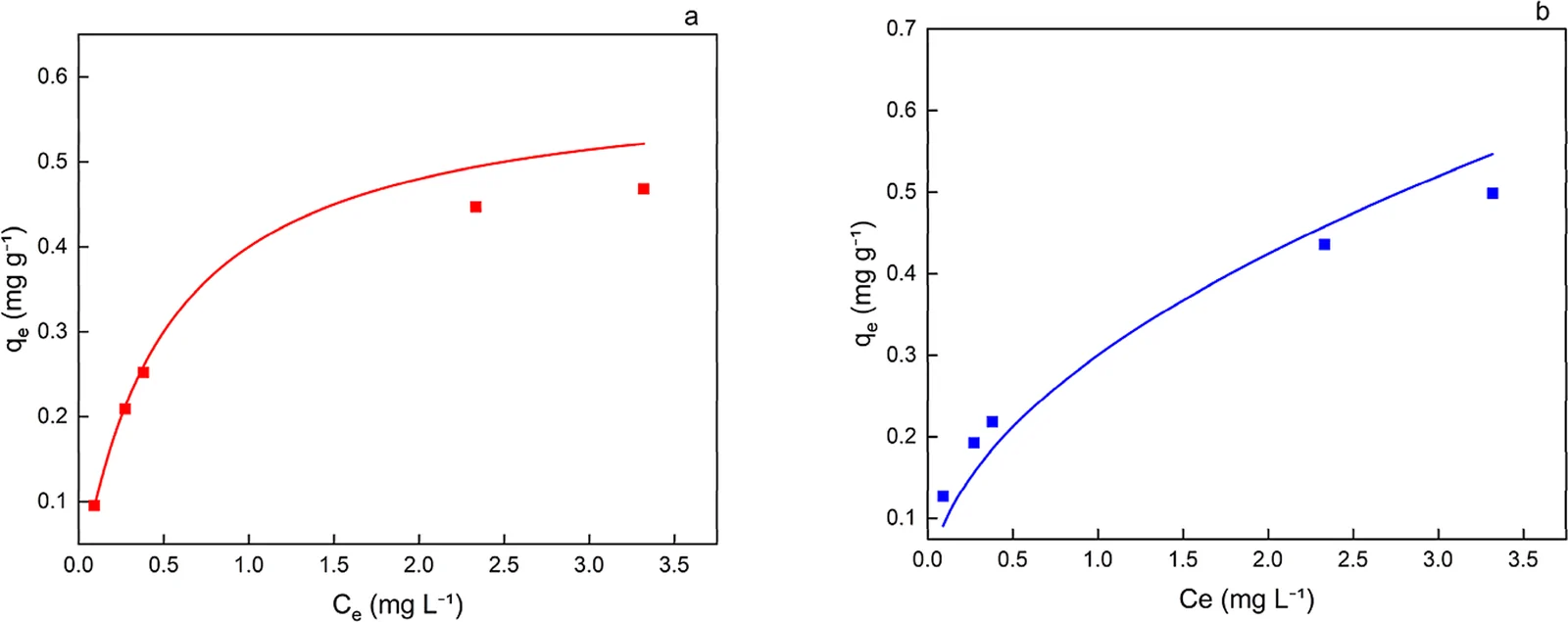
Nonlinear isotherm fitting of arsenate adsorption onto SBC/TiO_2_–CoFe_2_O_4_ composite at pH 8.5: (a) Langmuir model and (b) Freundlich model. Data points represent mean values of duplicate experiments (*n* = 2). Error bars (±SD) are smaller than the symbol size (mean RSD = 2.01%; full SD values are provided in Table S4). Experiments were conducted at an adsorbent dosage of 5.0 g L^–1^ (0.1 g in 20 mL solution), initial As­(V) concentrations of 0.5–6 mg L^–1^, and ambient temperature (25 ± 2 °C).

**Figure 10:**
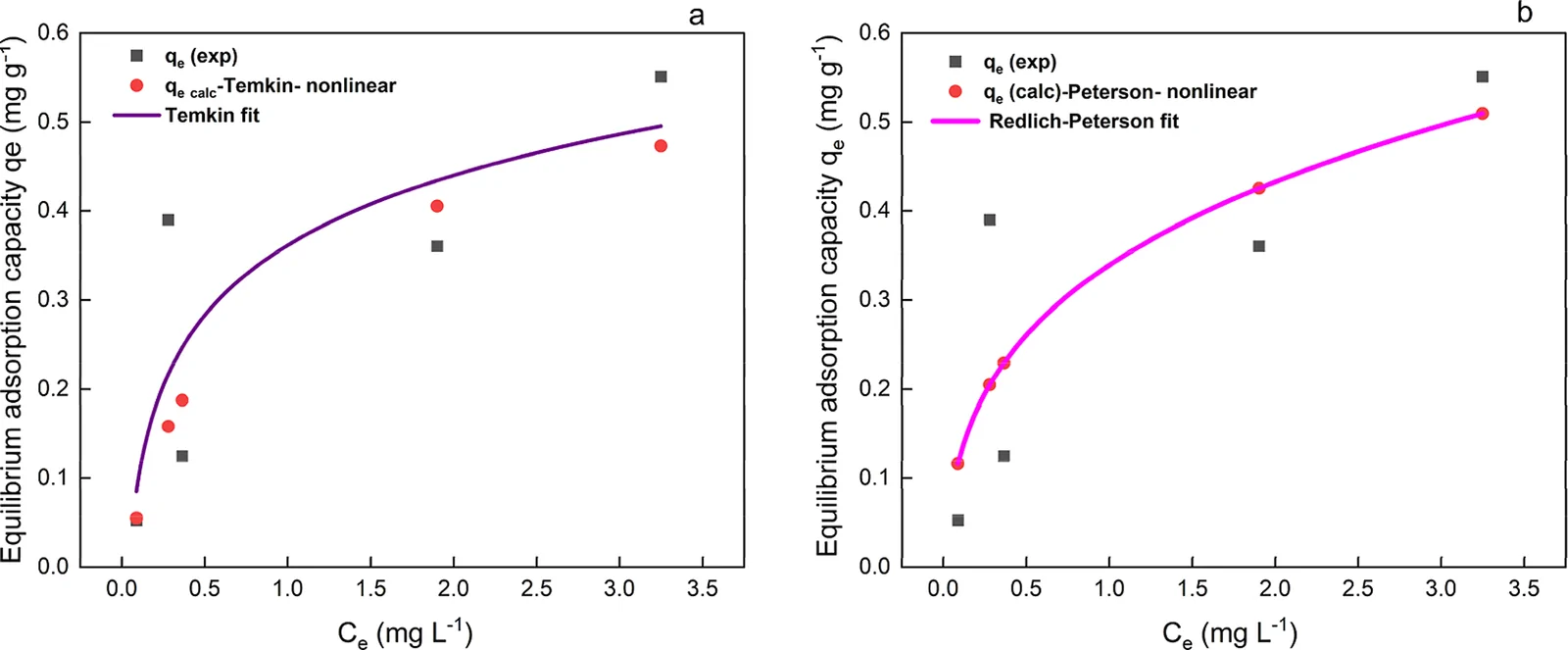
Nonlinear isotherm fitting of arsenate adsorption onto SBC/TiO_2_–CoFe_2_O_4_ at pH 8.5 using (a) Temkin and (b) Redlich–Peterson models. Black squares represent experimentally measured *q*_e_ (*n* = 2, mean ± SD, RSD = 2.01%; Tables S7 and S8), while red circles represent model-calculated values. Temkin: *B* = 0.1136, *A*_T_ = 24.08 L mg^–1^, *R* ^2^ = 0.673; Redlich–Peterson: *K*_R_ = 3.77 L g^–1^, β = 0.699, *R* ^2^ = 0.669.

**Table 4:** Equilibrium and Isotherm Adsorption Models (Langmuir and Freundlich) for As­(V) Adsorption with SBC/TiO_2_–CoFe_2_O_4_ ^a^

adsorbent	isotherm model	parameters	
SBC/TiO_2_–CoFe_2_O_4_	Langmuir pH 8.5	*K*_L_ (L mg^–1^)	2.422
		*q*_max_ (mg g^–1^)	0.526
		*R* ^2^	0.611
	Freundlich pH 8.5	*K*_f_ (mg g–1 (L mg^–1^)^1/*n*^)	0.316
		*n*	2.63
		*R* ^2^	0.635

a Parameters determined from duplicate experiments (*n* = 2); experiments were conducted at a fixed adsorbent dosage of 5.0 g L^–1^ (0.1 g adsorbent in 20 mL solution) in 50 mL Falcon tubes.

**Table 5:** Data Calculation of Error Indices^a^

model	*R* ^2^	RMSE	SSE	χ^2^	AIC
Langmuir	0.611	0.1146	0.0657	0.0657	–17.66
Freundlich	0.635	0.1110	0.0616	0.0616	–17.98
Temkin	0.673	0.1147	0.0657	0.0657	–17.67
Redlich–Peterson	0.669	0.1051	0.0552	0.0552	–18.32

a All models were fitted by nonlinear regression to the same experimental dataset (mean of duplicate measurements; *n* = 5 concentration levels; *C*_e_ = 0.092–3.32 mg L^–1^). χ^2^ was computed assuming unit variance (χ^2^ = SSE). $AIC=n.\ln\left(\frac{SSE}{n}\right)+2k$ , where *k* = number of fitted parameters (*k* = 2 for Langmuir, Freundlich, and Temkin; *k* = 3 for Redlich–Peterson). SSE values for Langmuir (0.06565) and Temkin (0.06572) are numerically close but distinct; both are rounded to 0.0657 in the table.

**Table 6:** Summary of Adsorption Isotherm Models Describing Arsenate Uptake on SBC/TiO_2_–CoFe_2_O_4_ at pH 8.5^a^

isotherm model	key parameters	*R* ^2^	interpretation at pH 8.5	dominant adsorption type	strengths/limitations with current dataset (*n* = 5)
**Langmuir**	*K*_L_ = 2.42 (L mg^–1^) *q*_max_ = 0.526 (mg g^–1^)	0.611	acceptable fit consistent with monolayer adsorption on relatively uniform metal-oxide domains (Fe^3+^, Ti^4+^); moderate *R* ^2^ reflects mechanistic complexity of the multi-phase surface	predominantly monolayer adsorption on energetically similar active sites	provides physically meaningful parameters (*q*_max_, *K*_L_); slightly lower statistical performance than other models; limited data points restrict robustness of homogeneity assumption
**Freundlich**	*K*_F_ = 0.316 ((mg g^–1^)(Lmg^–1^)^1^/^*n*^) *n* = 2.63	0.635	marginally improved empirical fit relative to Langmuir (ΔAIC = 0.32, below the threshold of 2 for meaningful distinction); *n* > 1 confirms favorable, cooperative adsorption on a surface characterized by non-uniform binding energies	multilayer adsorption on heterogeneous surface	best describes energetic heterogeneity empirically; lacks a theoretical saturation capacity; statistical improvement over Langmuir is modest given the small dataset
**Temkin**	*B* = 0.1136 *A*_T_ = 24.1 L mg^–1^	0.673	highest *R* ^2^ among two-parameter models; the Temkin assumption of a linearly decreasing heat of adsorption with surface loading is consistent with the energetically heterogeneous multi-phase surface comprising Fe-, Ti-, and biochar-associated domains of differing binding energy; parameter estimates carry non-trivial uncertainty given *n* = 5	heterogeneous energy distribution across binding sites	captures energetic heterogeneity explicitly; relatively wide confidence intervals due to *n* = 5; interpretation sensitive to limited concentration range
**Redlich-Peterson**	*K*_R_ = 3.77 L g^–1^ *a*_R_ = 10.11 (L mg^–1^) β = 0.6987	0.669	best overall statistical performance across all error metrics; β = 0.699 (95% CI: 0.45–0.95) excludes both 0 (pure Freundlich) and 1 (pure Langmuir), confirming intermediate adsorption behavior consistent with a composite surface comprising both relatively uniform metal-oxide sites and more energetically heterogeneous biochar domains	combined monolayer and heterogeneous multilayer tendencies	most flexible model; lowest SSE and AIC; three-parameter structure increases parameter uncertainty with small dataset; preferred model for heterogeneous composite systems

a *K*_L_ (Langmuir constant), *K*_R_ (Redlich-Peterson constant), *K*_F_ (Freundlich constant), *q*_max_ (Maximum adsorption capacity), Δ*H*_ads_) Heat of adsorption (*a*_R_ (Redlich-Peterson constant), β (Redlich-Peterson) (β = 1: pure Langmuir behavior; β = 0: pure Freundlich behavior), *B* (Temkin Constant). All parameters determined by nonlinear regression on the same experimental dataset (mean of duplicate measurements, *n* = 5).

Nonlinear fitting of the Langmuir model yielded a maximum monolayer adsorption capacity of *q*_max_ = 0.526 mg g^–1^ and a Langmuir affinity constant of *K*_L_ = 2.422 L mg^–1^, reflecting notable arsenate–surface affinity under the tested conditions (Table 4). The coefficient of determination (*R* ^2^ = 0.611) is moderate and warrants explicit mechanistic interpretation rather than dismissal as experimental inadequacy. At pH 8.5, arsenate exists predominantly as HAsO_4_^2–^ and engages the composite surface through at least three concurrent interaction pathways: outer-sphere electrostatic association at protonated metal hydroxyl groups, hypothesized inner-sphere ligand exchange at Fe^3+^ and Ti^4+^ sites (inferred from FTIR band shifts and literature precedent), and surface complexation at mixed-valence CoFe_2_O_4_ domains (Section 3.1). This mechanistic multiplicity means that no single two-parameter isotherm model is expected to capture the full energetic landscape with high *R* ^2^ fidelity; the moderate *R* ^2^ reflects an inherent limitation of classical isotherm theory applied to multi-mechanism heterogeneous systems, not a deficiency in data quality. Comparable *R* ^2^ ranges (0.58–0.71) have been reported for arsenate adsorption onto chemically analogous composite materials,^22^ consistent with this behavior being characteristic of similar heterogeneous composite systems. The Freundlich model produced marginally superior statistical metrics (*R* ^2^ = 0.635, SSE = 0.0616, RMSE = 0.1110, AIC = −17.98) compared to the Langmuir model (SSE = 0.0657, AIC = −17.66); however, the AIC difference (ΔAIC = 0.32) falls well below the threshold of 2 conventionally required to distinguish model performance, indicating statistically comparable descriptive adequacy.^61^ The Freundlich heterogeneity parameter *n* = 2.63 (>1) confirms favorable adsorption on a surface characterized by non-uniform binding energies, consistent with the composite’s mixed-phase architecture.

Additional mechanistic context was provided by applying the Temkin and Redlich–Peterson frameworks, both of which incorporate surface heterogeneity and adsorbate–adsorbent interaction energies. Nonlinear fitting of the Temkin model yielded an affinity parameter *A*_T_ = 24.08 L mg^–1^ and a Temkin constant *B* = 0.1136 (*R* ^2^ = 0.673, RMSE = 0.1147, χ^2^ = 0.0657; Table 5). Given the limited dataset (*n* = 5), these parameter estimates carry non-trivial uncertainty, consistent with a surface comprising energetically distinct Fe-, Ti-, and biochar-associated binding domains. The Redlich–Peterson model, which mathematically bridges Langmuir and Freundlich behavior, achieved the best overall statistical performance across all evaluated metrics: SSE = 0.0552, RMSE = 0.1051, χ^2^ = 0.0552, and AIC = −18.32 (Table 5). The heterogeneity parameter β = 0.699 (95% CI: 0.45–0.95) is the most mechanistically informative result of the isotherm analysis: its value, which excludes both 0 (pure Freundlich) and 1 (pure Langmuir), is consistent with intermediate adsorption behavior on a composite surface comprising both relatively uniform Fe- and Ti-centered oxide sites and more energetically heterogeneous biochar carbon domains. The affinity constant *K*_R_ = 3.77 L g^–1^ further reflects a substantial arsenate binding tendency of the SBC/TiO_2_–CoFe_2_O_4_ surface under the alkaline conditions studied. This heterogeneous-surface interpretation is further consistent with the non-monotonic *q*_e_ behavior observed in Table 1 (Section 3.2), where *q*_e_ decreased from 0.39 mg g^–1^ at *C*_0_ = 2 mg L^–1^ to 0.36 mg g^–1^ at *C*_0_ = 4 mg L^–1^ a pattern reproduced across duplicate measurements (RSD = 0.2%) and therefore attributable to genuine surface heterogeneity rather than experimental error.

Taken together, the convergence of all four isotherm models on a heterogeneous-surface interpretation, the moderate but data-consistent *R* ^2^ values, and the non-monotonic *q*_e_ feature discussed above collectively indicate that arsenate uptake occurs across an energetically diverse population of binding sites.

Taken together, the four isotherm models converge on a consistent interpretation: arsenate uptake on SBC/TiO_2_–CoFe_2_O_4_ is governed by a heterogeneous population of binding sites of differing energies, arising from the composite’s mixed-phase architecture comprising Fe- and Ti-centered metal oxide domains and energetically more diverse biochar carbon surfaces. This interpretation is consistent with, but not uniquely demonstrated by, the isotherm data alone; unambiguous assignment of specific binding modes to specific site populations would require complementary spectroscopic evidence (e.g., XPS or surface complexation modeling) beyond the scope of the present study. The moderate *R* ^2^ values (0.611–0.673) are accordingly interpreted as a reflection of genuine surface complexity rather than experimental inadequacy, a conclusion reinforced by cross-framework consistency with the PSO kinetic model and the Boyd film diffusion analysis.

The thermodynamic feasibility of arsenate adsorption onto the SBC/TiO_2_–CoFe_2_O_4_ composite was investigated at 298, 308, and 318 K under optimized conditions (initial As­(V) = 2 mg L^–1^, pH 8.5, adsorbent dosage = 5.0 g L^–1^). The standard Gibbs free energy (Δ*G*°), enthalpy (Δ*H*°), and entropy (Δ*S*°) changes were derived from the van’t Hoff equation using the dimensionless equilibrium constant K°, where *K*_L_ (L mg^–1^) is the Langmuir affinity constant and *M*_w_ = 74,920 mg mol^–1^ is the molar mass of arsenate, as described in Section 2.5.2.^62,63^ The resulting thermodynamic parameters are summarized in Table 7.

**Table 7:** Thermodynamic Parameters for SBC/TiO_2_–CoFe_2_O_4_ for Adsorption Experiment of As­(V) (*n* = 2)

thermodynamic adsorbent	temperature (K)	*K*_l_	Δ*G*° (kJ mol^–1^)	Δ*Η*° (kJ mol^–1^)	Δ*S*° (J mol^–1^ K^–1^)	*R* ^2^
SBC/TiO_2_–CoFe_2_O_4_	298	17768.9 ± 18	–24.24 ± 0.44	41.92 ± 5.1	221.75 ± 16.3	0.987
	308	27723.4 ± 26	–26.20 ± 0.47			
	318	51621.3 ± 41	–28.69 ± 0.52			

The negative Δ*G*° values at all three temperatures (−24.24 ± 0.44, −26.20 ± 0.47, and −28.69 ± 0.52 kJ mol^–1^ at 298, 308, and 318 K, respectively) confirm spontaneous and thermodynamically favorable arsenate uptake across the studied temperature range. The progressive shift toward more negative Δ*G*° with increasing temperature indicates enhanced thermodynamic driving force at elevated temperatures, consistent with chemisorption-dominated processes (|Δ*G*°| = 20–80 kJ mol^–1^).^64^

The positive Δ*H*° (+41.92 ± 5.1 kJ mol^–1^) confirms an endothermic adsorption process, reflecting the enthalpic cost of displacing coordinated water molecules from the metal oxide surface hydroxyl groups and the hydrated arsenate anion. The magnitude of Δ*H*° is characteristic of thermally activated chemisorption involving Fe–O–As and Ti–O–As bond formation, consistent with values reported for arsenate binding at iron and titanium oxide surfaces,^65^ and accounts for the observed enhancement in removal efficiency with increasing temperature.

The large positive Δ*S*° (+221.75 ± 16.3 J mol^–1^ K^–1^) reflects a significant gain in interfacial disorder driven by two concurrent processes: release of structured water molecules from metal oxide hydration shells upon ligand exchange, and displacement of surface-bound OH^–^ by the bulkier HAsO_4_^2–^, both of which increase translational freedom in the interfacial layer. Thermodynamic decomposition confirms that the entropic contribution (*T*Δ*S*° = 66.08 kJ mol^–1^ at 298 K) substantially exceeds the enthalpic penalty (Δ*H*° = +41.92 kJ mol^–1^), identifying the process as entropy-driven at all studied temperatures.

The convergence of negative Δ*G*°, positive Δ*H*°, and large positive Δ*S*° defines a thermodynamic signature consistent with the mechanistic framework established by the kinetic and isotherm analyses: PSO-dominated chemisorption (χ^2^ = 0.067), heterogeneous binding reflected by the Redlich–Peterson β parameter (0.699), and ligand-exchange-controlled surface reactivity indicated by Boyd and FTIR analyses. This cross-framework consistency supports thermally activated, entropy-favored adsorption at metal oxide domains, interpreted as a plausible mechanistic hypothesis supported by convergent indirect evidence across kinetic, isotherm, and thermodynamic frameworks; direct confirmation requires XPS or EXAFS characterization.

### 3.4 Reusability and Stability

Four consecutive adsorption–desorption cycles were conducted to evaluate the reusability of the SBC/TiO_2_–CoFe_2_O_4_ composite under optimal conditions (pH 8.5, initial As­(V) concentration = 2 mg L^–1^). After each adsorption cycle, regeneration was performed by rinsing the exhausted adsorbent with 5% HCl (v/v), followed by washing with deionized water and oven drying prior to reuse. As depicted in Figure 11, the first cycle exhibited a removal efficiency of 85.36%, consistent with the initial performance observed during the zero-cycle experiment. Removal efficiency decreased progressively in subsequent cycles, reaching 70.08%, 54.27%, and 52.39% in the second, third, and fourth cycles, respectively, representing a cumulative decline of approximately 33 percentage points across four full regeneration cycles.

**Figure 11:**
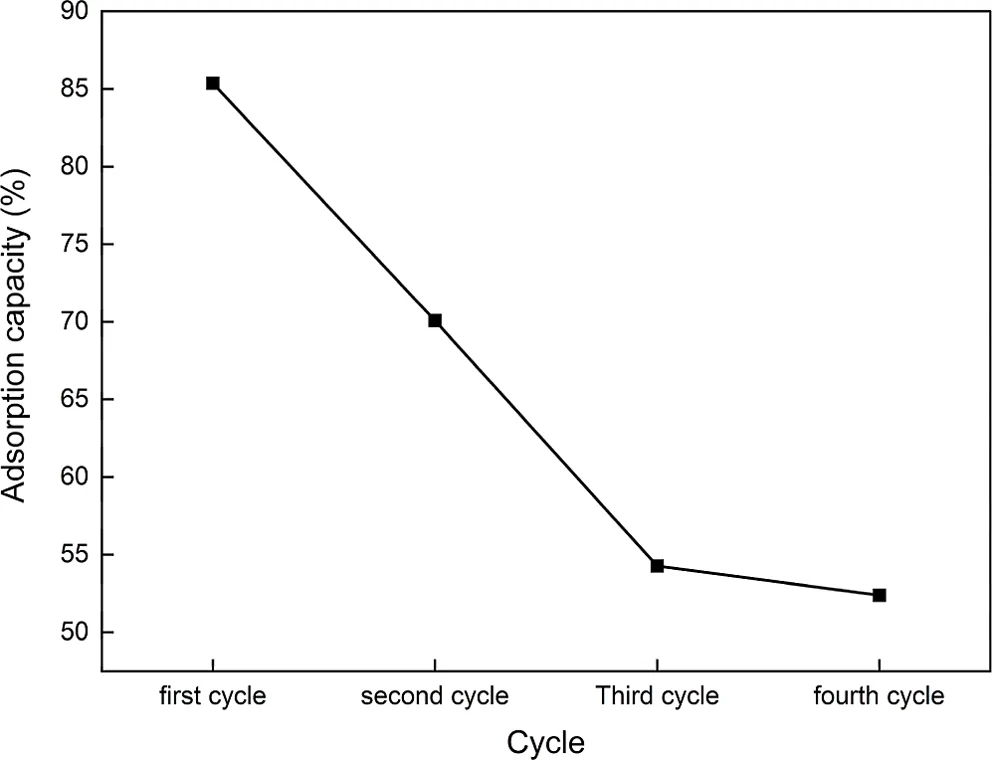
Reusability of SBC/TiO_2_–CoFe_2_O_4_.

The selection of 5% HCl as the regenerating agent was mechanistically motivated: under acidic conditions, surface hydroxyl groups on Fe^3+^ and Ti^4+^ sites undergo protonation, substantially reducing electrostatic affinity for anionic HAsO_4_^2–^ species and facilitating competitive desorption via H^+^-mediated ligand exchange.^66,67^ This mechanism is well-established for iron- and titanium-based oxide adsorbents and was preferred over alkaline regeneration (e.g., NaOH) because hydroxide-based desorption, while effective for arsenate displacement via OH^–^ competition, simultaneously promotes dissolution of CoFe_2_O_4_ spinel domains and irreversible modification of TiO_2_ surface charge distribution under strongly alkaline conditions effects that accelerate composite degradation over repeated cycles.^68^

To evaluate the chemical stability of the composite during repeated regeneration, the concentrations of Fe, Co, and Ti released into the 5% HCl regenerant solution were quantified by ICP-OES after each adsorption–desorption cycle (Supporting Information Table S9). As shown in Table S9, Fe concentrations in the spent regenerant increased from 0.18 mg L^–1^ after cycle 1 to 0.54 mg L^–1^ after cycle 4, while Co and Ti concentrations increased from 0.07 to 0.31 mg L^–1^ and from 0.02 to 0.22 mg L^–1^, respectively. Despite this gradual increase with cycle number, the corresponding leaching percentages remained exceptionally low throughout below 0.050% for Fe, below 0.055% for Co, and below 0.017% for Ti across all four regeneration cycles. These values confirm that the TiO_2_ and CoFe_2_O_4_ phases remained strongly immobilized on the biochar matrix and that the composite retained high structural integrity under repeated acidic regeneration conditions.

The progressive reduction in removal efficiency across cycles therefore reflects the combined effects of partial active-site deactivation and protonation-induced restructuring of surface hydroxyl environments, rather than wholesale structural degradation of the metal oxide framework. The exceptionally low leaching percentages (≤0.055% for all metals across all cycles; Table S9) confirm that dissolution-driven compositional loss was negligible, and that the gradual performance decline is more plausibly attributable to progressive reduction in accessible high-affinity binding site density consistent with the biphasic deactivation kinetics reported for analogous composite adsorbents.^69^ This interpretation is further supported by the marked deceleration in performance loss between cycles 3 and 4 (54.27% → 52.39%, a drop of only 1.88 percentage points), suggesting that the composite approaches a performance plateau at which the remaining active sites exhibit sufficient chemical stability to resist further acid-induced deactivation.

Although direct post-regeneration structural characterization (FTIR, SEM) was not performed, the combined evidence of low metal leaching and decelerating performance decline provides indirect support for the structural integrity of the composite after four regeneration cycles. Direct post-regeneration structural characterization is identified as a priority for future work to confirm this inference. The observed retention of greater than 50% removal efficiency after four complete regeneration cycles is consistent with, and in several cases superior to, performance trends reported for chemically analogous composite adsorbents in the peer-reviewed literature: Navarathna et al.^22^ reported a decline from ∼80% to ∼50% for magnetic biochar composites over four HCl-regenerated cycles; Chen et al.^70^ reported ∼78% to ∼45% over five cycles for Fe_3_O_4_/biochar systems; and Shirinkar et al.^71^ demonstrated that an SBC/MIL-101­(Fe) composite retained approximately 50% of its initial arsenic removal efficiency after four HCl-regenerated cycles. In each case, comparable or greater performance losses were considered acceptable for environmental remediation applications. In the present system, a residual removal efficiency of 52.39% at cycle 4 continues to represent effective arsenate capture at an initial concentration of 2 mg L^–1^, reducing solution-phase As­(V) to near the WHO provisional guideline value of 10 μg L^–1^ the primary operational benchmark for this application domain.^24,72^

The experimentally realized adsorption capacity (*q*_e_ = 0.39 mg g^–1^ at *C*_0_ = 2 mg L^–1^) and the Langmuir-derived maximum capacity (*q*_max_ = 0.526 mg g^–1^), together with the documented four-cycle regeneration profile and confirmed low metal leaching, are contextualized relative to representative biochar- and metal-oxide-based arsenate adsorbents in Supporting Information Table S2. While the *q*_max_ of the present composite falls within the lower portion of the reported range, direct numerical comparison across studies is constrained by substantial differences in initial arsenic concentration (*C*_0_ ranging from 0.1 to >100 mg L^–1^ among the compiled comparators); as adsorption isotherms are inherently nonlinear, capacities obtained near isotherm saturation cannot be meaningfully compared with those obtained in the Henry’s law region. The present composite’s contribution is therefore best assessed through its combination of attributes: evaluation under environmentally representative dilute conditions (*C*_0_ = 2 mg L^–1^, pH 8.5), magnetic recoverability enabling facile separation without filtration infrastructure, a quantified four-cycle regeneration profile with measurably decelerating performance decline, and confirmed low metal leaching (≤0.055%) across all regeneration cycles a combination of operational attributes not jointly present in any single comparator study identified in Supporting Information Table S2.

Although performance declined across cycles, the SBC/TiO_2_–CoFe_2_O_4_ composite demonstrated meaningful retention of arsenate adsorption capacity over repeated regeneration, demonstrating laboratory-scale reusability under synthetic matrix conditions. Future investigations should evaluate alternative regenerating agents such as dilute NaOH, Na_2_HPO_4_, and NaHCO_3_ to assess whether milder desorption conditions can reduce cumulative surface damage while maintaining adequate desorption efficiency, thereby optimizing the balance between regenerative capacity and long-term material stability. The regeneration behavior observed in this study reflects realistic operational conditions rather than idealized permanent stability, providing a laboratory-scale evaluation of the composite’s suitability for repeated adsorption applications.

#### 3.4.1 Economic Feasibility and Practical Implications

A preliminary cost analysis was conducted to assess the economic position of the SBC/TiO_2_–CoFe_2_O_4_ composite relative to commercially available arsenic adsorbents (Tables S10–S12). All cost estimates are based on 2024–2025 bulk market prices at ≥1 kg purchase scale, with full assumptions documented in the Supporting Information (Table S10). The estimated production cost of 35.9–40.3 USD kg^–1^ is dominated by nano-TiO_2_ and cobalt precursors (∼85–90% of raw material cost); sugarcane bagasse biochar contributes only 1–3%. Processing and energy expenses add 3.5–5.5 USD kg^–1^.

At the optimized operating conditions (dosage = 5.0 g L^–1^, *C*_0_ = 2 mg L^–1^, pH 8.5), the single-use treatment cost is 0.092–0.103 USD per mg As removed, based on the experimentally determined adsorption capacity of 0.39 mg g^–1^. A transparent comparison with Table S10 reveals that this cost is higher than those reported for granular ferric hydroxide (0.0008–0.012 USD mg^–1^), industrial activated carbon (0.0025–0.03 USD mg^–1^), and Fe/Al-impregnated AC (0.003–0.04 USD mg^–1^). This cost differential is acknowledged explicitly and reflects two compounding factors: the high unit cost of nano-TiO_2_ and cobalt precursors, and the modest realized capacity under the dilute test conditions employed.

Three contextual considerations are essential for interpreting this differential. First, the superior adsorption capacities reported for commercial adsorbents in Tables S10 and S11 were largely determined at arsenic concentrations of 10–100 mg L^–1^ conditions that approach isotherm saturation and systematically overstate performance relative to environmentally realistic dilute scenarios (*C*_0_ = 1–5 mg L^–1^) where GFH and activated carbon operate in their own low-capacity Henry’s law regimes.^70^ A capacity-normalized comparison conducted under identical dilute conditions would substantially narrow the observed cost gap. Second, the magnetic recoverability of SBC/TiO_2_–CoFe_2_O_4_ eliminates filtration and separation infrastructure costs that add significantly to the operational expenditure of non-magnetic adsorbents in field-scale systems, a value not captured in the raw cost-*per*-mg metric. It should be noted, however, that field-scale magnetic separation requires dedicated infrastructure such as high-gradient magnetic separators (HGMS), whose capital and operational costs are not incorporated in the present laboratory-scale estimates; at laboratory scale, separation was achieved using a hand-held NdFeB permanent magnet at negligible cost (∼5–20 USD). A preliminary estimation of pilot-scale HGMS infrastructure costs, based on published equipment costs for small-scale units (capacity: ∼100 L h^–1^; capital cost: ∼2000–5000 USD; electrical energy consumption: ∼0.5–2.0 kWh m^–3^), suggests an additional infrastructure contribution of approximately 0.003–0.008 USD per mg As removed under the test conditions of this study; full details of this estimation are provided in Supporting Information Section S5. Adding this infrastructure component to the composite’s four-cycle regenerated cost (0.046 USD mg^–1^; Table S12) yields an adjusted pilot-scale estimate of approximately 0.049–0.054 USD mg^–1^, which remains within the upper range of Fe/Al-impregnated activated carbon (0.003–0.04 USD mg^–1^). These estimates carry significant uncertainty, and a formal pilot-scale TEA is required for definitive assessment. Third, incorporating four magnetic regeneration cycles reduces the effective treatment cost from 0.113 USD mg^–1^ (single-use) to 0.069, 0.059, and 0.046 USD mg^–1^ after two, three, and four cycles, respectively a cumulative 59% reduction that substantially improves the economic profile over the material’s operational lifetime.

A formal sensitivity analysis quantifies the pathway to economic competitiveness as a function of adsorption capacity (Table S11). The cost–capacity relationship is strictly inverse and proportional: realizing the theoretical Langmuir maximum (*q*_max_ = 0.526 mg g^–1^) reduces cost by 26% to 0.071 USD mg^–1^; a 1.9-fold capacity increase (*q*_e_ ∼0.75 mg g^–1^, achievable through mild pH or temperature optimization) reaches 0.05 USD mg^–1^; and a 2-fold increase achieves 0.048 USD mg^–1^, within the upper range of commercial Fe/Al-AC (0.003–0.04 USD mg^–1^). These projections define concrete and experimentally achievable performance targets for future material optimization and identify operating condition refinement as the primary lever for closing the economic gap with established commercial adsorbents.

All cost estimates presented here are explicitly scoped as laboratory-scale, order-of-magnitude feasibility assessments and should not be interpreted as industrial deployment forecasts. The pilot-scale HGMS infrastructure cost estimation provided above (Supporting Information Section S5 and S5.1) represents an order-of-magnitude projection only and is subject to substantial uncertainty depending on site-specific energy costs, equipment procurement, maintenance schedules, and regulatory compliance requirements. A rigorous techno-economic assessment (TEA) at pilot scale incorporating process intensification, energy recovery, full magnetic separation infrastructure amortization, waste management, and bulk procurement economics is required to validate industrial viability and is identified as a necessary next step for translational development of this composite.

In summary, while the current cost-*per*-mg metric of the SBC/TiO_2_–CoFe_2_O_4_ composite exceeds that of conventional commercial adsorbents under single-use conditions, the analysis identifies a well-defined and achievable improvement trajectory: four regeneration cycles independently reduce the effective cost to 0.046 USD mg^–1^ (0.049–0.054 USD mg^–1^ including estimated pilot-scale HGMS infrastructure), and a sub-2-fold capacity enhancement through operating condition optimization further improves economic positioning. The composite’s distinctive combination of magnetic recoverability, multi-cycle reusability, and targeted performance under environmentally relevant dilute conditions defines a differentiated value proposition for decentralized or resource-constrained arsenic remediation scenarios where infrastructure simplicity and magnetic separation offer operational advantages not captured in raw material cost comparisons.

### 3.5 Effect of Competing Phosphate on Arsenate Removal

To partially address the influence of competing anions under conditions more representative of natural groundwater chemistry, the effect of phosphate (PO_4_^3–^) the most aggressive known competitor for arsenate binding sites on metal-oxide surfaces was evaluated at environmentally relevant concentrations (0, 2, 5, and 10 mg L^–1^) under otherwise identical experimental conditions (*C*_0_(As­(V)) = 2 mg L^–1^, pH 8.5, adsorbent dose = 5.0 g L^–1^, contact time = 24 h, *n* = 2).

As shown in Table 8, arsenate removal efficiency decreased progressively with increasing phosphate concentration, from 86.7 ± 0.2% in the absence of phosphate to 81.0 ± 0.1%, 70.9 ± 0.1%, and 44.6 ± 0.1% at phosphate concentrations of 2, 5, and 10 mg L^–1^, respectively. The low standard deviations across all conditions (≤0.2%) confirm the reproducibility of the competitive adsorption measurements. At the lowest phosphate concentration tested (2 mg L^–1^, corresponding to a PO_4_:As molar ratio of approximately 0.65:1), removal efficiency remained above 81%, indicating that the composite retains substantial arsenate uptake capacity under moderate competitive conditions. At 5 mg L^–1^ phosphate (PO_4_:As molar ratio ≈1.6:1), removal declined by approximately 16 percentage points relative to the control, consistent with partial site competition between structurally analogous tetrahedral oxyanions HAsO_4_^2–^ and HPO_4_^2–^ for Fe^3+^ and Ti^4+^ surface hydroxyl groups. At the highest concentration tested (10 mg L^–1^; PO_4_:As molar ratio ≈3.2:1), removal efficiency declined to 44.6 ± 0.1%, reflecting substantial competitive inhibition under conditions that markedly exceed typical groundwater phosphate levels. These results are consistent with the inner-sphere complexation hypothesis proposed in Section 3.1: both arsenate and phosphate form structurally analogous bidentate binuclear surface complexes at metal-oxide hydroxyl sites through ligand exchange, and therefore compete directly for the same high-affinity binding domains on Fe^3+^ and Ti^4+^ active sites. The observed concentration-dependent inhibition pattern is therefore expected and has been documented for structurally analogous iron- and titanium-oxide-based adsorbents.^72,73^

**Table 8:** Effect of Phosphate (PO_4_^3–^) Concentration on Arsenate Removal Efficiency by SBC/TiO_2_–CoFe_2_O_4_ ^a^

PO_4_^3–^ (mg L^–1^)	As(V) removal efficiency (%)	absolute reduction (%)
0 (control)	86.7 ± 0.2	—
2	81.0 ± 0.1	5.66
5	70.9 ± 0.1	15.80
10	44.6 ± 0.1	42.10

a Conditions: *C*_0_(As­(V)) = 2 mg L^–1^, pH 8.5, adsorbent dose = 5.0 g L^–1^, contact time = 24 h, *T* = 25 ± 2 °C, *n* = 2; values reported as mean ± SD.

Importantly, naturally occurring phosphate concentrations in arsenic-contaminated groundwater typically range from 0.1 to 2 mg L^–1^, and the 2 mg L^–1^ condition tested here therefore represents the most environmentally relevant competitive scenario, at which the composite retained 81.0 ± 0.1% arsenate removal efficiency. This result indicates that the composite maintains functionally meaningful arsenate uptake capacity under groundwater-realistic phosphate competition, while acknowledging that performance under higher-phosphate conditions (e.g., agricultural or eutrophic settings) would be substantially reduced. Evaluation of additional competing species including silicate, sulfate, bicarbonate, and natural organic matter remains an identified priority for follow-up investigation prior to field deployment.

## 4 Conclusion

The SBC/TiO_2_–CoFe_2_O_4_ nanocomposite achieved an experimental arsenate adsorption capacity of 0.39 mg g^–1^ at the environmentally relevant test condition (*C*_0_ = 2 mg L^–1^, pH 8.5), designated as the primary performance metric of this study; the corresponding Langmuir-derived theoretical maximum (*q*_max_ = 0.526 mg g^–1^) is reported separately for isotherm characterization and literature benchmarking purposes (Supporting Information Table S2). The composite further exhibited removal efficiency exceeding 52% after four magnetic regeneration cycles and a 59% reduction in effective treatment cost relative to single-use operation. The principal contribution of this work is the systematic characterization of arsenate adsorption by an SBC/TiO_2_–CoFe_2_O_4_ composite under environmentally realistic dilute alkaline conditions not previously investigated for this system. The convergence of PSO-dominated kinetics (χ^2^ = 0.067), Redlich–Peterson isotherm behavior (β = 0.699), and entropy-driven thermodynamic spontaneity (Δ*G*° = −24.24 to −28.69 kJ mol^–1^) is consistent with a chemisorption-dominated uptake pathway, consistent with a chemisorption-dominated uptake pathway, with inner-sphere complexation at Fe^3+^ and Ti^4+^ sites proposed as a plausible mechanism based on the available indirect evidence; definitive confirmation would require complementary characterization such as XPS or EXAFS.

The composite’s functional profile moderate adsorption capacity paired with magnetic recoverability and multi-cycle regenerability defines a differentiated platform for decentralized arsenate remediation, where operational simplicity and magnetic separation outweigh unit-capacity maximization, although the realized cost-*per*-mg-As-removed (0.092–0.103 USD mg^–1^, narrowing to 0.046 USD mg^–1^ at four cycles) currently exceeds that of established commercial adsorbents under equivalent conditions. ICP-OES quantification of Fe, Co, and Ti in the spent regenerant confirmed negligible metal leaching across all four regeneration cycles (≤0.050% for Fe, ≤0.055% for Co, ≤0.017% for Ti), demonstrating high chemical stability of the composite framework under repeated acidic regeneration conditions. Systematic evaluation under real groundwater matrices, combined with continuous-flow packed-bed experiments and extended regeneration cycling, will determine whether the multifunctional design strategy embodied by SBC/TiO_2_–CoFe_2_O_4_ translates into practically deployable and economically viable arsenic remediation at scale.

### 4.1 Limitations and Future Work

Fundamental limitations of this study include evaluation conducted primarily under synthetic single-solute matrix conditions; while competitive adsorption experiments with phosphate confirmed retained arsenate removal efficiency of 81.04 ± 0.1% at environmentally representative phosphate concentrations (2 mg L^–1^ PO_4_^3–^), the influence of silicate, sulfate, bicarbonate, natural organic matter, and mixed-speciation systems remains to be established, without controlled evaluation of background ionic strength effects independent of specific anion competition, and without assessment of As­(III) or mixed-speciation systems, as evaluation was restricted to As­(V). Additional limitations include laboratory-scale cost projections, five-point isotherm characterization, regeneration testing limited to four cycles, evaluation restricted to the 0.5–6 mg L^–1^ range rather than WHO-relevant μg L^–1^ residual concentrations, absence of direct experimental comparison against single-oxide (TiO_2_-only or CoFe_2_O_4_-only) binary control composites synthesized and tested under identical conditions, and absence of BET surface area, X-ray diffraction (XRD), vibrating-sample magnetometry (VSM), and X-ray photoelectron spectroscopy (XPS) characterization due to instrumental accessibility constraints during the study period. Although duplicate measurements (*n* = 2) demonstrated acceptable reproducibility (RSD: 1–3.6%), the limited number of replicates reduces statistical power; triplicate repetition of key experiments is identified as a priority for follow-up work. Systematic evaluation under real groundwater matrices and competitive ion conditions, combined with continuous-flow packed-bed experiments and extended regeneration cycling, will determine whether the multifunctional design strategy embodied by SBC/TiO_2_–CoFe_2_O_4_ translates into practically deployable and economically viable arsenic remediation at scale.

## Supplementary Material



## Notes

Additional experimental detail and results about Effect of Initial Solution pH and Point of Zero Charge (PZC) and Economic Feasibility and Sustainability Assessment (PDF)
