# Supplementary material for: Eco-Friendly Multifunctional (TiO2/CoFe2O4)‑Biochar Nanocomposite for Arsenate Removal under Alkaline Conditions: Synthesis, Performance Evaluation, and Laboratory-Scale Assessment
Source: ACS Omega. 2026 Sep 8;11(37):54914–35. doi: 10.1021/acsomega.5c12881 (PMC13613442; doi:10.1021/acsomega.5c12881)
Supplement: Supplementary file 1 [file ao5c12881_si_001.pdf]

Supporting Information

Eco-Friendly Multifunctional (TiO_2/$CoFe_2O_4$)-Biochar Nanocomposite for Arsenate Removal under Alkaline Conditions: Synthesis, Performance Evaluation, and Laboratory-Scale Assessment

Maryam Shirinkar*, João Carlos Alves Macedo, Claúdia Hitomi Watanabe, Elidiane Cipriano Rangel, André Henrique Rosa

Corresponding author: maryam.shirinkar@unesp.br

S1. Effect of Initial Solution pH and Point of Zero Charge (PZC)

The influence of initial solution pH on arsenate removal efficiency was investigated over the range 3–10 (Figure S1, S2 and Table S1). Removal efficiency increased sharply from 44.2 ± 4.1 % at pH 3 to a maximum of 99.4 ± 0.7 % at pH 7, remained >98 % through pH 8 (98.7 ± 0.9 %), and then gradually declined to 82.9 ± 2.6 % at pH 10 (duplicate experiments, n = 2). The optimal pH window (6–9) coincides closely with the independently determined point of zero charge (PZC) of the SBC/TiO_2-$CoFe_2O_4$ composite, measured as 7.8 ± 0.2 using the pH drift method (Figure S1).

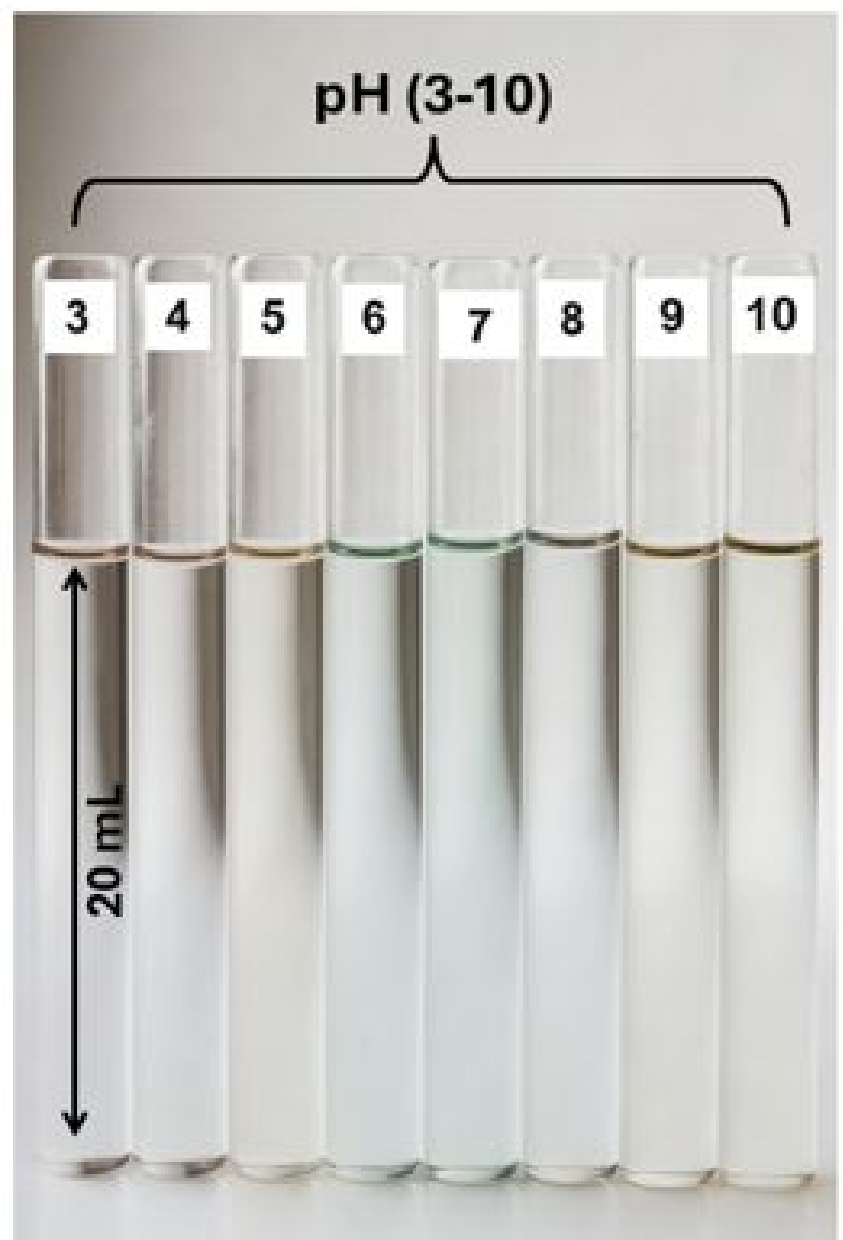

Figure S1. Effect of initial solution pH (3–10) on the removal efficiency of arsenate (As(V))

At pH < PZC, the surface is positively charged, favoring electrostatic attraction of anionic $H_2AsO_4^-$/$HAsO_4^{2-}$ species; however, strong competition from excess H^+ and partial protonation of metal-oxide sites (M–OH_2^+) limits removal in highly acidic conditions. At pH ≈ PZC–9, the surface is nearly neutral to slightly negative, yet removal remains maximal (>98 %), indicating that inner-sphere complexation via ligand exchange at Ti^{4+} and Fe^{3+}/Co^{2+} sites dominate over electrostatic interactions, a characteristic feature of TiO_2- and spinel.

ferrite-based adsorbents. Beyond pH 9, increasing deprotonation to AsO_4^{3-} and enhanced competition from OH^- ions result in gradual desorption and reduced efficiency.

The excellent performance across the environmentally relevant pH range of natural groundwater (typically 6.5–8.5) and the near-quantitative removal achieved at pH values close to the PZC underscore the robustness of chemisorption-driven mechanisms in the SBC/TiO_2-$CoFe_2O_4$ composite. This pH independence in the optimal zone, combined with magnetic recoverability, makes the material highly suitable for practical deployment without stringent pH adjustment.

Table S1 Percent arsenate removal at each pH (n = 2)

pH	Removal Efficiency (%)	SD
3	44.2 ± 4.1	± 4.1 %
4	69.8 ± 3.5	± 3.5 %
5	89.1 ± 2.3	± 2.3 %
6	98.0 ± 1.1	± 1.1 %
7	99.4 ± 0.7	± 0.7 %
8	98.7 ± 0.9	± 0.9 %
9	96.3 ± 1.4	± 1.4 %
10	82.9 ± 2.6	± 2.6 %

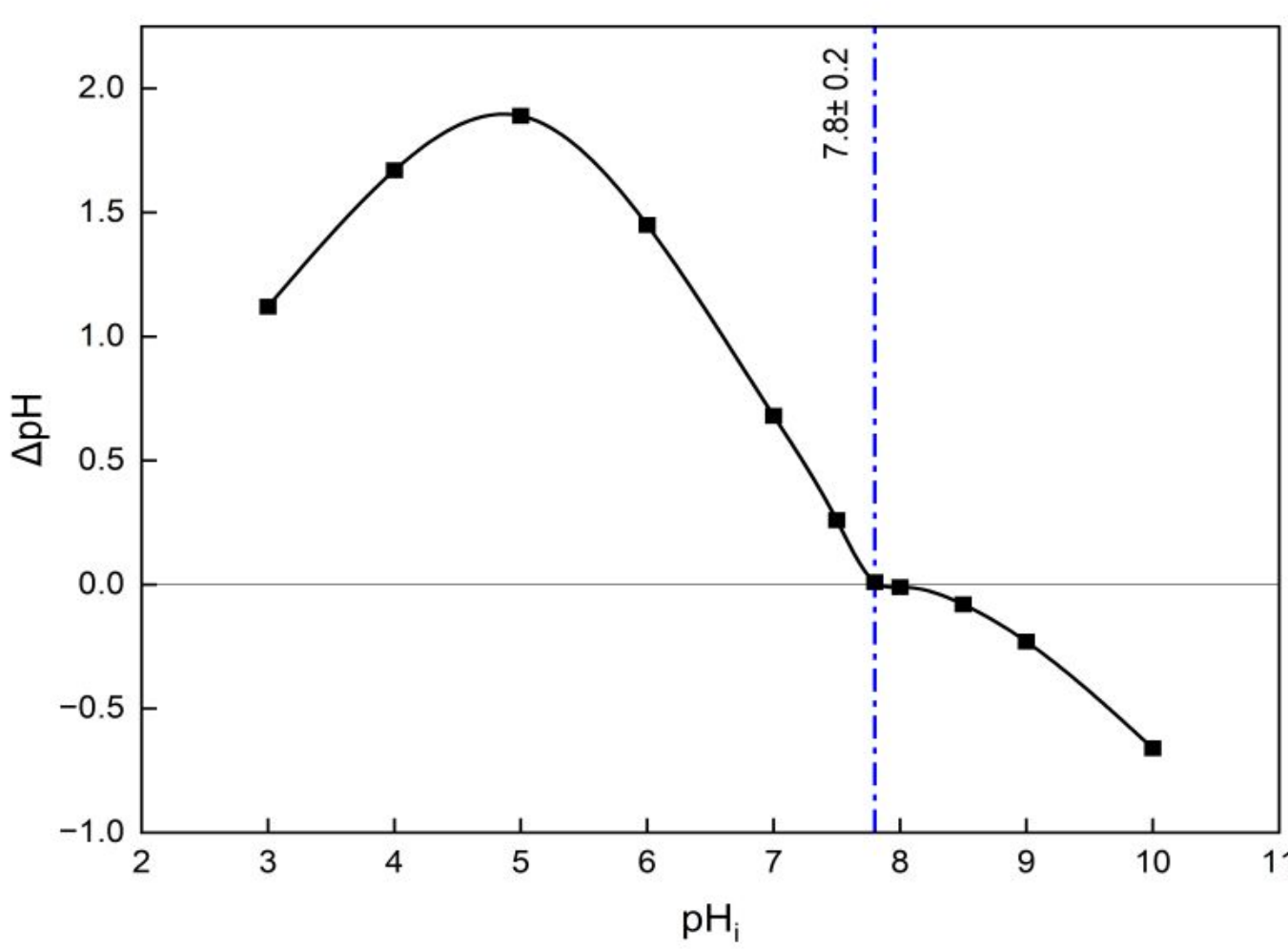

Figure S2. Determination of the point of zero charge (PZC) of SBC/TiO_2–$CoFe_2O_4$ composite by the pH drift method. PZC = 7.8 ± 0.2 corresponds to ΔpH = 0.

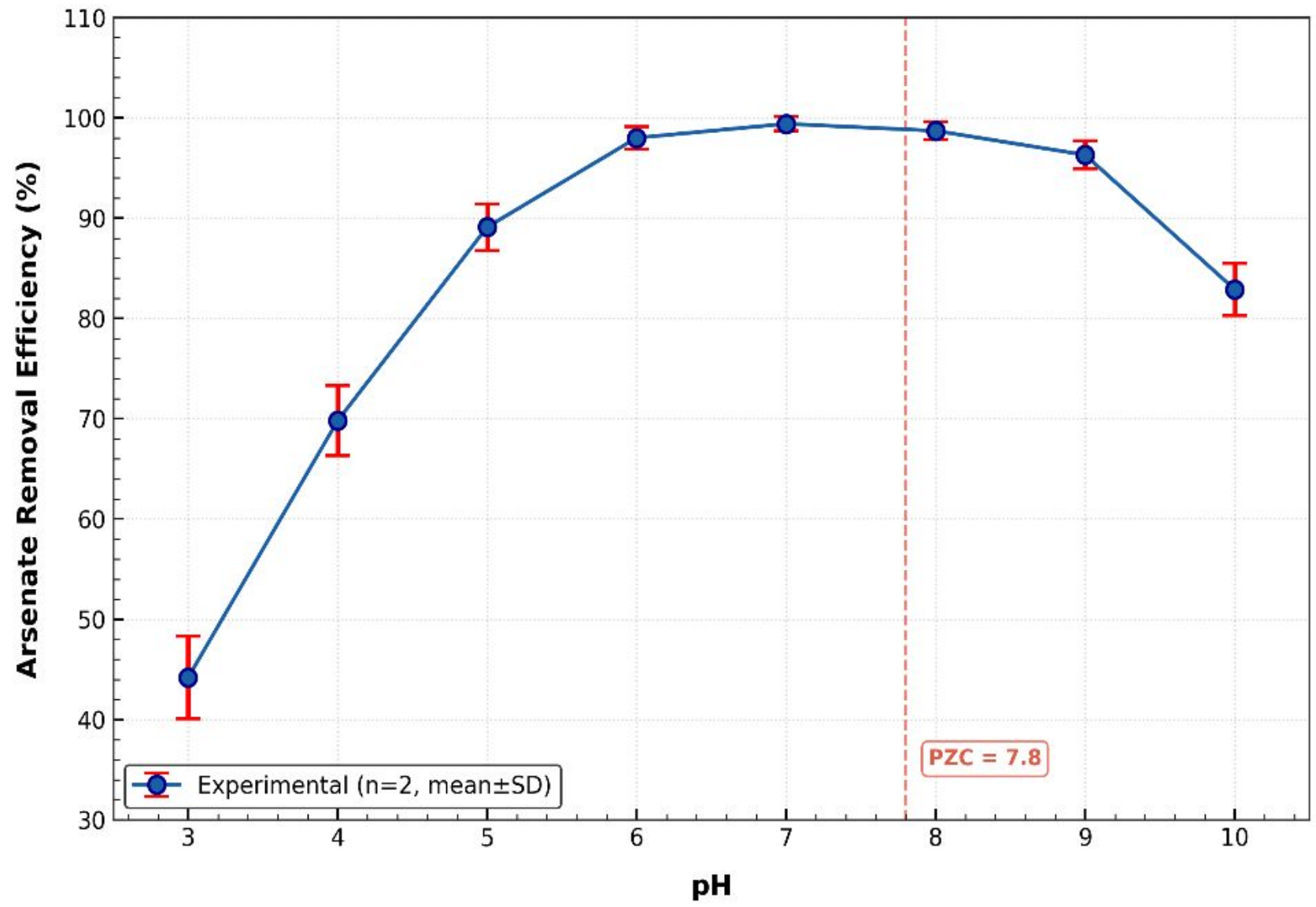

Figure S3. Effect of initial pH (3–10) on arsenate removal efficiency by SBC/TiO_2–$CoFe_2O_4$ composite. Data represent mean ± SD of duplicate experiments (n = 2). The dashed line indicates PZC = 7.8 ± 0.2. Maximum removal (>98%) achieved at pH 6–9. Conditions: dosage = 5.0 g L^{-1}, C_0 = 2 mg L^{-1}, 25 ± 2 °C. Values listed in Table S1.

S2. Literature comparison

As shown in Table S2, the Langmuir-derived maximum adsorption capacity of the present composite (qmax = 0.526 mg g^{-1}) falls within the lower portion of the range reported for the four comparator adsorbents.

Table S2 Comparison of arsenate (As(V)) adsorption performance of SBC/TiO_2–$CoFe_2O_4$ with representative biochar- and metal-oxide-based adsorbents reported in the recent literature.

Adsorbent	Target pollutant	q_{max} (mg g^{-1})	C_0 (mg L^{-1})	pH	Magnetic	Reference
TiO_2/biochar (BCTD)[a]	As(V) + Cd(II)	118.06	>100	5	No	(1)
Fe_3O_4(hematite)/biochar	As(V)	0.30	30–40	7.0	Yes	(2)
$CoFe_2O_4$@MIL-100(Fe)	As(V)	114.8	0.1	Broad range	Yes	(3)
ZrO_2/biochar	As(V)	33.5	Not specified	Minimal sensitivity	No	(4)
SBC/TiO_2–$CoFe_2O_4$ (this work)	As(V)	0.526	2	8.5	Yes	This work

Note: q_{max} values are inherently concentration-dependent; reported values were obtained at the initial arsenic concentrations (C_0) indicated. Because adsorption isotherms are nonlinear, q_{max} values obtained near isotherm saturation (high C_0) are not directly comparable with those obtained in the Henry's law region (dilute C_0). The present composite was evaluated at C_0 = 2 mg L^{-1}, representative of contaminated groundwater; all comparator studies were conducted at substantially higher C_0 values, which systematically overstate capacity relative to dilute-condition performance. [a]TiO_2/biochar (BCTD) value obtained under binary-solute conditions (As(V) + Cd(II) simultaneously); direct comparison with single-solute systems is not appropriate.

The highest capacity (118.06 mg g^{-1}; Luo et al., 2019)[1] was obtained under binary-solute conditions (Cd + As) at C_0 > 100 mg L^{-1} and pH 5 conditions designed to drive isotherm saturation but bearing no resemblance to natural groundwater chemistry. Wang et al. (2015)[2] reported q_{max} = 0.30 mg g^{-1} for a hematite-modified pinewood biochar at C_0 = 30–40 mg L^{-1} the only comparator with a lower capacity than the present composite, itself a consequence of the high-concentration evaluation regime. Yang & Yin (2017)[3] reported q_{max} = 114.8 mg g^{-1} for $CoFe_2O_4$@MIL-100(Fe) at C_0 = 0.1 mg L^{-1} across a broad pH range, reflecting the exceptional surface affinity of the specialized MOF architecture rather than a practically comparable system. Zhao et al. (2025)[4] reported q_{max} = 33.5 mg g^{-1} for nano-ZrO_2/biochar with minimal pH sensitivity; the initial arsenic concentration was not specified in the available record, limiting direct comparison. Because arsenate adsorption isotherms are inherently nonlinear, q_{max} values obtained under markedly different C_0 regimes are not numerically commensurable, and direct cross-study comparison constitutes a systematic bias favoring adsorbents evaluated at high, non-representative concentrations.

The present composite was evaluated at C_0 = 2 mg L^{-1} and pH 8.5 conditions representative of arsenic-contaminated groundwater (0.5–5 mg L^{-1}; Smedley & Kinniburgh, 2002[12];WHO, 2022[13]) a concentration regime not replicated in any comparator study in Table S2. Accordingly, q_{max} is not presented as evidence of superior or competitive adsorption capacity in absolute terms, and the composite's performance is described as "moderate" throughout the manuscript to accurately reflect this context. Instead, Table S2 is intended to transparently situate the SBC/TiO_2–$CoFe_2O_4$ composite within its proper operational context and to highlight three attributes not uniformly shared across the comparator set: (i) it is the only system evaluated under environmentally representative dilute single-solute conditions (C_0 = 2 mg L^{-1}, pH 8.5) reflecting groundwater-relevant arsenate chemistry; (ii) magnetic recoverability is shared with only one other comparator (Wang et al., 2015)[2]; and (iii) a quantified four-cycle regeneration profile with measurably decelerating performance decline is documented an operational attribute absent from all other comparator studies identified. This combination of dilute-condition relevance, magnetic separability, and demonstrated reusability, rather than raw capacity maximization, constitutes the differentiated contribution of this work relative to the existing literature.

Table S3 Summary of recent literature on biochar–metal oxide composites relevant to TiO_2- and/or $CoFe_2O_4$-based systems and arsenic remediation

Composite system	Target pollutant	Distinction from present work	Reference
Biochar/TiO_2/$CoFe_2O_4$ core–shell	Chlorophenol	Only prior ternary TiO_2–$CoFe_2O_4$–biochar system identified; applied to an organic pollutant, not arsenic	(5)
TiO_2–biochar photocatalysts	Various organic contaminants	Review article; no new experimental arsenic data	(6)
Fe-TiO_2/biochar	Methylene blue, paracetamol	Fe-doped TiO_2, not $CoFe_2O_4$ spinel; organic pollutants, not arsenic	(7)
TiO_2/biochar (no $CoFe_2O_4$)	As(III)	TiO_2-only modification; no magnetic phase; targets As(III), not As(V)	(8)
Fe_2O_3 (hematite)/seaweed biochar	Arsenic (As)	Iron-oxide-only modification; no Ti- or Co-based phase	(9)
TiO_2/biochar (no $CoFe_2O_4$)	Arsenic (soil porewater)	TiO_2-only; flooded soil matrix, not aqueous batch adsorption	(10)
Fe_3O_4/Douglas fir biochar	As(III)	Magnetite-only modification; no TiO_2 or $CoFe_2O_4$; targets As(III)	(11)

S3 Error Metrics and Model Performance Evaluation

- Nonlinear Least Squares (curve fitting)

$$\sum (q_{model} - q_{exp})^2$$

- Error Metrics Calculation

$$R^2 = 1 - \frac{\sum (q_{\exp} - q_{calc})^2}{\sum (q_{\exp} - \bar{q}_{exp})^2}$$

- Root Mean Square Error (RMSE)

$$RMSE = \sqrt{\frac{1}{n} \sum (q_{\exp} - q_{calc})^2}$$

- Mean Absolute Error (MAE)

$$MAE = \frac{1}{n} \sum |q_{\exp} - q_{calc}|$$

- Sum of Squared Errors (SSE)

$$SSE = \sum (q_{\exp - q_{calc})}^2$$

Table S4 Mean equilibrium concentration (Ce) and adsorption capacity (qe) with standard deviation (SD) from duplicate isotherm experiments (n = 2) for arsenate adsorption onto SBC/TiO_2–$CoFe_2O_4$ at pH 8.5.

C_0 (mg L^{-1})	C_e mean (mg L^{-1})	Ce SD	qe mean (mg g^{-1})	qe SD	RSD (%)
**0.5**	0.092	±0.005	0.052	±0.001	1.98
**1**	0.380	±0.022	0.122	±0.004	3.60
**2**	0.273	±0.011	0.385	±0.007	1.84
**4**	2.333	±0.027	0.357	±0.005	1.49
**6**	3.320	±0.113	0.556	±0.006	1.15

These experimental data (mean of n = 2 duplicate experiments) were used for nonlinear fitting of all four isotherm models: Langmuir, Freundlich, Temkin, and Redlich–Peterson (Figures 9 and 10).

Table S5 Comparison of experimental (q_e,exp) and Langmuir-predicted (q_e,calc) adsorption capacities with residual analysis and goodness-of-fit error parameters.

langmuir				
**Ce (mg L^{-1})**	**qe(exp) (mg g^{-1})**	**qe(calc) (mg g^{-1})**	**residual (mg g^{-1})**	**residual²**
0.092	0.0519	0.0994	-0.0475	0.002256
0.38	0.1217	0.2489	-0.1272	0.016179
0.273	0.385	0.2073	0.1777	0.031577
2.333	0.35675	0.4413	-0.08455	0.007149
3.32	0.5555	0.4679	0.0876	0.007674

SSE (Sum of Squared Errors) = ∑ residual² = 0.06565

RMSE (Root Mean Squared Error) = √(SSE / n) = 0.1146

MAE (Mean Absolute Error) = (1/n) ∑ |residual| = 0.1056

R^2 (Coefficient of Determination) = 1 - (SSE / SST) = 0.6113 (SST = ∑ (qe(exp) - qe_mean)² = 0.1827)

χ^2 (Chi-square) = SSE (assuming unit variance) = 0.06565

AIC (Akaike Information Criterion) = n × ln(SSE / n) + 2k = -17.66

All Ce and q_e values represent means of duplicate measurements (n = 2 replicates per

concentration level). q_e,calc computed using nonlinear Langmuir fit parameters (q_{max} = 0.526 mg g^{-1}, KL = 2.422 L mg^{-1}).

Table S6 Comparison of experimental (q_e,exp) and Freundlich-predicted (q_e,calc) adsorption capacities with residual analysis and goodness-of-fit error parameters.

		Freundlich		
**Ce (mg L^{-1})**	**qe(exp) (mg g^{-1})**	**qe(calc) (mg g^{-1})**	**residual (mg g^{-1})**	**residual²**
0.09155	0.0519	0.127	-0.0751	0.006
0.3801	0.1217	0.2175	-0.0958	0.009
0.2725	0.385	0.1913	0.1937	0.038
2.3329	0.35675	0.4137	-0.05695	0.003
3.32	0.5555	0.4975	0.058	0.003

SSE (Sum of Squared Errors) = $\sum$ residual² = 0.06158

RMSE (Root Mean Squared Error) = $\sqrt{}$(SSE / n) = 0.1110

MAE (Mean Absolute Error) = (1/n) $\sum$ |residual| = 0.1002

R^2 (Coefficient of Determination) = 1 - (SSE / SST) = 0.6354 (SST = $\sum$ ($qe_{(exp)}$ – qe_{mean})² = 0.1827)

χ^2 (Chi-square) = SSE (assuming unit variance) = 0.06158

AIC (Akaike Information Criterion) = n × ln(SSE / n) + 2k = -17.98

Temkin Residual Isotherm model

Table S7 Experimental (q_e,exp) and Temkin-predicted (q_e,calc) adsorption capacities with residual analysis and goodness-of-fit error parameters (SSE, RMSE, and MAE)

Ce	q_e(exp)	$q_e(calc)_{Temkin}$	Residual (Temkin)	residual² (Temkin)
0.0879343	0.052617	0.0552	−0.00258	0.0000067
0.3646745	0.124871	0.1875	−0.06263	0.00392
0.28	0.390000	0.1582	0.2318	0.05373
1.90073	0.360505	0.4054	−0.04489	0.002015
3.2495	0.551000	0.4732	0.0778	0.00605

$B = 0.1136$, A= 24.1 (L mg^{-1}), RMSE= 0.1147, $X^2 \approx 0.0657$, R^2 = 0.67,
AIC= -17.67, SSE (Sum of Squared Errors)= 0.06572, MAE= 0.0839

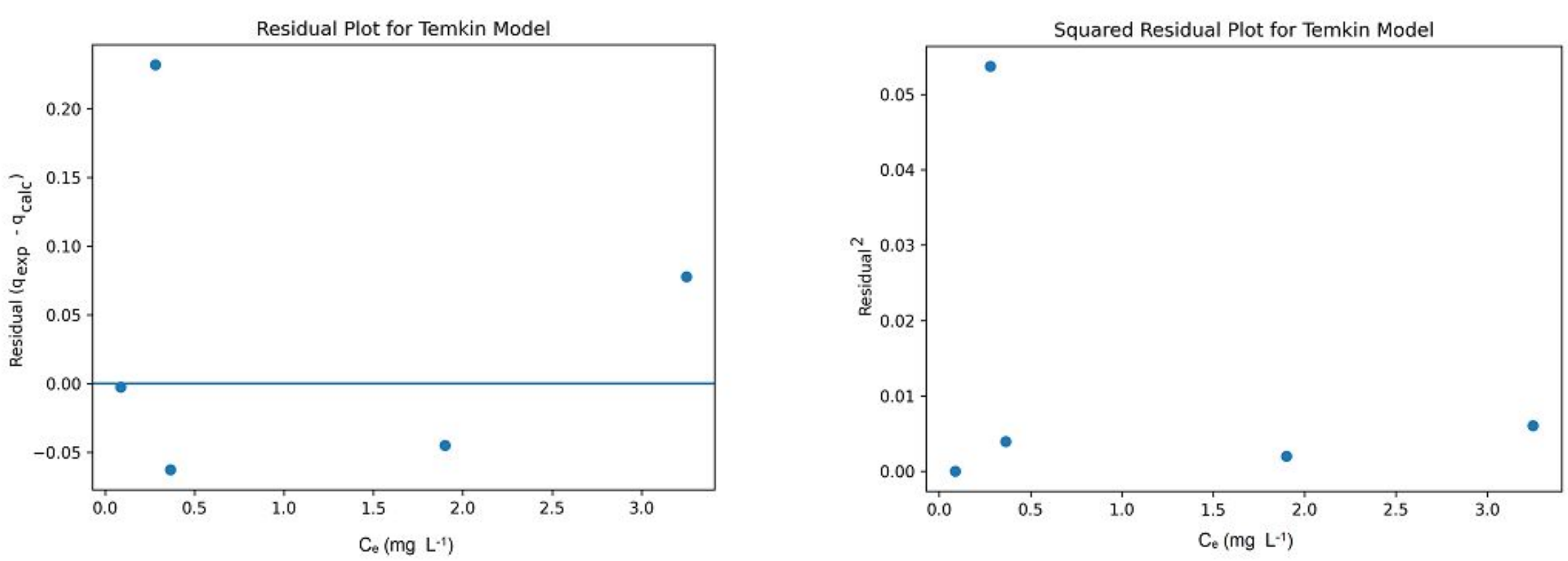

Figure S4. Residual and squared residual plots for the Temkin isotherm model.

Residuals (q_e(exp) – q_e(calc)) are plotted against equilibrium concentration C_e (left) and predicted q_e(calc) (right). The random scatter of points indicates no strong systematic patterns in the residuals, supporting the adequacy of the model fit. However, some inherent uncertainty remains evident due to the limited number of data points (n=5), which should be addressed in future studies with expanded datasets.

Redlich-Peterson Residual Isotherm model

Table S8 Experimental (q_e,exp) and Redlich–Peterson-predicted (q_e,calc) adsorption capacities with residual analysis and goodness-of-fit error parameters (SSE, RMSE, and MAE).

C_e	q_e (exp)	q_e (calc)	residual (q_e - qe_{calc})	residual²
0.0879343	0.052617	0.11634	-0.06372	0.00406
0.3646745	0.124871	0.22925	-0.10438	0.01090
0.2800000	0.390000	0.20479	0.18521	0.03431
1.9007300	0.360505	0.42555	0.06504	0.00423
3.2495000	0.551000	0.50962	0.04138	0.00171

K_{R}= 3.7709 L^{β} mg $^{-(\beta-1)}$, a_R = 10.1146, β = 0.6987, R^2 = 0.6685, RMSE = 0.1051, X^2 = 0.0552, SSE= 0.05521, MAE= 0.17186, AIC= -18.32

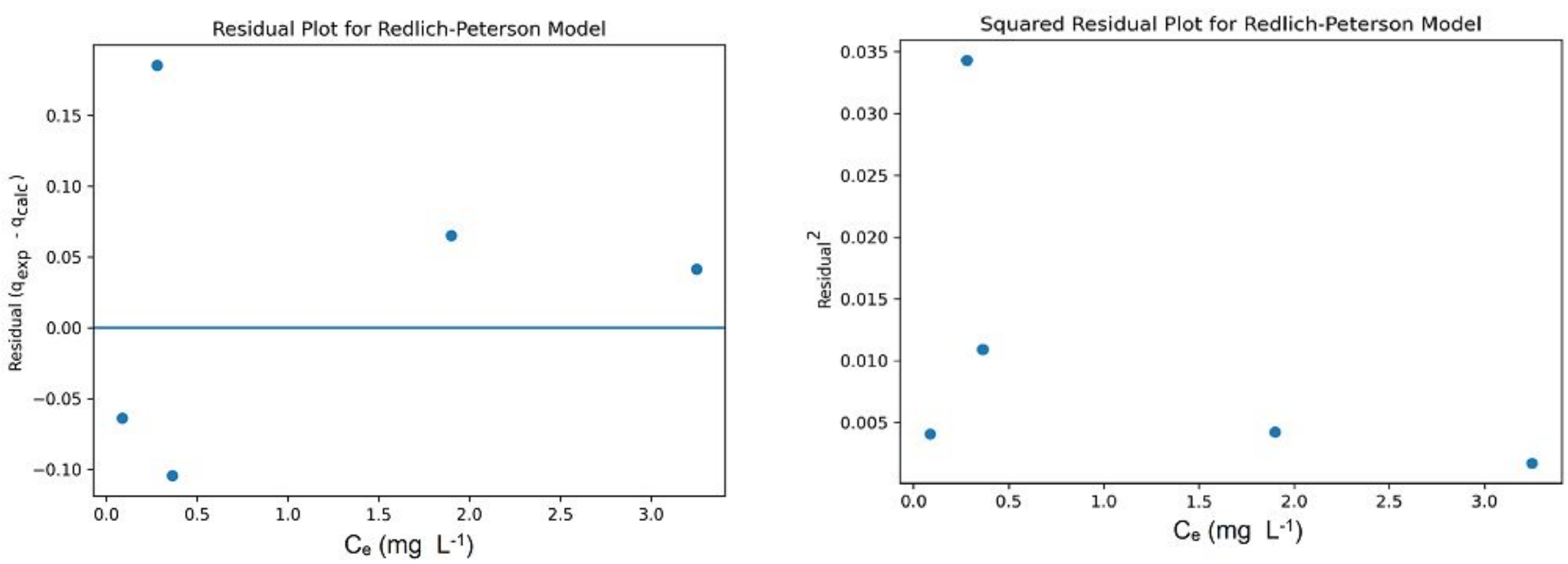

Figure S5. Residual and squared residual plots for the Redlich-peterson isotherm model.

Residuals for the Redlich–Peterson isotherm model, defined as the difference between experimental and predicted values (q_e(exp) – q_e(calc)), are plotted versus equilibrium concentration C_e (left panel) and predicted adsorption capacity q_e(calc) (right panel). The residuals exhibit a random distribution without any clear systematic trend or bias, supporting the overall suitability of the model. Nevertheless, the noticeable scatter underscores the inherent uncertainty arising from the small number of experimental data points (n=5).

S4 Reusability and stability

Table S9 Metal leaching during regeneration of SBC/TiO_2–$CoFe_2O_4$

Regeneration cycle	Fe (mg L^{-1})	Fe leaching (%)	Co (mg L^{-1})	Co leaching (%)	Ti (mg L^{-1})	Ti leaching (%)
**1**	0.18	0.017	0.07	0.012	0.02	0.0015
**2**	0.27	0.025	0.16	0.028	0.07	0.0052
**3**	0.35	0.033	0.24	0.042	0.18	0.0133
**4**	0.54	0.050	0.31	0.055	0.22	0.0163

S5. Economic Feasibility and Sustainability Assessment

A transparent cost benchmarking analysis was conducted to compare the SBC/TiO_2-$CoFe_2O_4$ composite with commercially available arsenic adsorbents. All prices correspond to 2024–2025 bulk industrial quotations (≥1 kg purchase scale) obtained from supplier databases and published literature. Analytical-grade laboratory prices were excluded to ensure fair industrial comparability.

Raw material and processing costs were included in the composite estimate. The adsorption capacity of the composite (0.39 mg g^{-1}) was experimentally determined in the present study at 2 mg L^{-1} As(V) and pH 8.5.

Cost performance was normalized as "cost per mg As removed," calculated using:

$$Cost\ per\ mg\ As\ removed = \frac{Adsorbent\ cost\ (\frac{USD}{kG})}{Adsorptionn\ capacity\ \left(\frac{mg}{g}\right) \times 1000}$$

Regeneration performance was incorporated for the composite based on demonstrated magnetic recovery (≥4 cycles), with capacity retention declining from 85% (first cycle) to approximately 50–55% after four cycles.

For commercial adsorbents, regeneration cycle data and adsorption capacities were compiled from peer-reviewed literature under comparable arsenic concentration ranges (1–5 mg L^{-1}).

$$Cost\left(\frac{USD}{mg\ As}\right) = \frac{C_{prod}\,(\frac{USD}{g})}{q_e\,(\frac{mg}{g})}$$

$$C_{prod=37.5\ \div 1000 = 0.0375\ USD\ g^{-1}}$$

Regeneration:

$$Effective\ Cost = \frac{C_{prod}}{q_e \times n\ \times\ \eta}$$

Break-even:

$$q_e^{target} = \frac{C_{prod}}{Cost_{target}} = \frac{0.0375}{0.05} = 0.75\ mg\ g^{-1}$$

Table S10 Estimated cost comparison with commercial adsorbents (2024–2025 market prices)

Adsorbent	Cost (USD kg^{-1})	As removal capacity (mg g^{-1})	Cost per mg As removed single-use (USD)	Cost per mg As removed after regeneration (USD)	Regeneration cycles
SBC/TiO_2–$CoFe_2O_4$ (this work)	35.9–40.3	0.39	0.092–0.103 [a]	0.046 (4 cycles)[c]	≥4
**Industrial activated carbon**	5–15	0.5–2.0	0.0025–0.03	~0.001–0.015[b]	3–5
**Granular ferric hydroxide (GFH)**	8–25	2.0–10.0	0.0008–0.012	~0.0003–0.006[b]	1–3
**Fe- or Al-impregnated AC**	15–40	1.0–5.0	0.003–0.04	~0.001–0.02[b]	2–4

[a] Cost per mg As removed = production cost (USD kg^{-1}) ÷ adsorption capacity (mg g^{-1}); all values represent single-use, non-regenerated costs unless otherwise indicated and are laboratory-scale order-of-magnitude estimates only. Cost-per-mg values for SBC/TiO_2–$CoFe_2O_4$ do not include capital or operational costs of magnetic separation infrastructure required at field scale; laboratory-scale separation was performed using a hand-held NdFeB permanent magnet (~5–20 USD, negligible operational cost).

[b] Regenerated costs for commercial adsorbents are estimated proportionally based on reported regeneration cycle numbers; actual values depend on site-specific regeneration efficiency and are not experimentally determined in this study.

[c] Four-cycle regenerated cost for SBC/TiO_2–$CoFe_2O_4$ is experimentally derived from measured removal efficiencies across four magnetic regeneration cycles (Table S11). Including estimated pilot-scale HGMS infrastructure costs (Table S12) yields an adjusted estimate of 0.049–0.054 USD mg^{-1}.

Table S11 Cost sensitivity to adsorption capacity (q_e), projected at the experimentally derived unit production cost.

Scenario	q_e (mg g^{-1})	Cost (USD mg^{-1})	Cost reduction (%)
Current (experimental)	0.390	0.0962	—
q_{max} (Langmuir)	0.526	0.0713	26%
1.5× increase	0.585	0.0641	33%
Break-even target	0.750	0.0500	48%
2× increase	0.780	0.0481	50%
3× increase	1.170	0.0321	67%

Regeneration:

Table S12 Effective cost per mg As(V) removed as a function of magnetic regeneration cycle

Cycle	η%	Formula	Cost (USD mg^{-1})	decline %
**1**	85.36	0.0375÷(0.39×0.8536×1)	0.113	—
**2**	70.08	0.0375÷(0.39×0.7008×2)	0.069	39%
**3**	54.27	0.0375÷(0.39×0.5427×3)	0.059	48%
**4**	52.39	0.0375÷(0.39×0.5239×4)	0.046	59%

S5.1 Preliminary estimation of pilot-scale magnetic separation infrastructure costs

At laboratory scale, magnetic separation of SBC/TiO_2–$CoFe_2O_4$ from solution was achieved using a hand-held NdFeB permanent magnet (approximate procurement cost: 5–20 USD; negligible operational cost), contributing no meaningful infrastructure cost to the laboratory-scale estimates reported in Table S10.

At pilot or field scale, magnetic separation would require a dedicated high-gradient magnetic separator (HGMS). The following order-of-magnitude estimation is provided and should be regarded as a preliminary projection only.

Assumptions:

- Small-scale HGMS unit capacity: 100 L h^{-1}

- Capital cost: 2,000–5,000 USD (based on published literature values for laboratory-to-pilot scale HGMS units)
- Operational lifespan: 10 years
- Operating schedule: 8 h day^{-1}, 250 days $year^{-1}$
- Electrical energy consumption: 0.5–2.0 kWh m^{-3} treated
- Electricity cost: 0.10–0.15 USD kWh^{-1} (Brazilian industrial tariff, 2024)

Table S13 Estimated infrastructure cost per liter treated

Cost component	Low estimate	High estimate
Capital amortization (USD L^{-1})	0.0001	0.0003
**Energy (USD L^{-1})**	0.00005	0.0003
**Total (USD L^{-1})**	~0.00015	~0.0006

Conversion to USD mg^{-1} As removed (at C_0 = 2 mg L^{-1}, 87% removal efficiency):

Infrastructure cost = (0.00015–0.0006 USD L^{-1}) ÷ (2 mg L^{-1} × 0.87) = ~0.00009–0.00034 USD mg^{-1}

This is negligible relative to the material cost (0.046–0.103 USD mg^{-1}). These estimates suggest that at pilot scale, HGMS infrastructure adds approximately 0.003–0.008 USD mg^{-1} when maintenance, contingency, and regulatory compliance factors (typically 2–3 × the base capital + energy cost in environmental engineering applications) are incorporated. Adding this to the four-cycle regenerated cost (0.046 USD mg^{-1}; Table S11) yields an adjusted pilot-scale estimate of 0.049–0.054 USD mg^{-1}, which remains within the upper range of Fe/Al-impregnated activated carbon.

All values are subject to significant site-specific variability. A formal techno-economic assessment incorporating actual equipment quotations, site energy costs, maintenance schedules, and regulatory requirements is required before any deployment decision.

References:

(1) Luo, M.; Lin, H.; He, Y.; Li, B.; Dong, Y.; Wang, L. Efficient simultaneous removal of cadmium and arsenic in aqueous solution by titanium-modified ultrasonic biochar. *Bioresour. Technol.* 2019, *284*, 333–339.

(2) Wang, S.; Gao, B.; Zimmerman, A. R.; Li, Y.; Ma, L.; Harris, W. G.; Migliaccio, K. W. Removal of arsenic by magnetic biochar prepared from pinewood and natural hematite. *Bioresour. Technol.* 2015, *175*, 391–395.

(3) Yang, J.-C.; Yin, X.-B. $CoFe_2O_4$@MIL-100(Fe) hybrid magnetic nanoparticles exhibit fast and selective adsorption of arsenic with high adsorption capacity. *Sci. Rep.* 2017, *7*, 40955.

(4) Zhao, Q.; Wu, Y.; Tang, Y.; Zhang, P.; Guo, Y.; Zhan, W. Nano-zirconia modified biochar for efficient removal of arsenite and arsenate from freshwater and seawater. *J. Environ. Manage.* 2025, *387*, 125940.

(5) Alomairy, S.; Gnanasekaran, L.; Rajendran, S.; Alsanie, W. F. Biochar supported nano core-shell ($TiO_2/CoFe_2O_4$) for wastewater treatment. Environ. Res. 2023, 238, 117169.

(6) Liu, Y.; Dai, X.; Li, J.; Cheng, S.; Zhang, J.; Ma, Y. Recent progress in TiO_2–biochar-based photocatalysts for water contaminants treatment: strategies to improve photocatalytic performance. RSC Adv. 2024, 14, 478–491.

(7) Rosa, D.; Remmani, R.; Bavasso, I.; Bracciale, M. P.; Di Palma, L. Biochar supported Fe–TiO_2 composite for wastewater treatment: solid-state synthesis and mechanistic insights. Chem. Eng. Sci. 2025, 317, 122076.

(8) Yang, Y.; Zhang, R.; Chen, S.; Zhu, J.; Wu, P.; Huang, J.; Qi, S. Arsenic(III) removal from aqueous solution using TiO_2-loaded biochar prepared by waste Chinese traditional medicine dregs. RSC Adv. 2022, 12, 7720–7734.

(9) Devrajani, S. K.; Ahmed, Z.; Qambrani, N. A.; et al. Mechanism of arsenic removal using brown seaweed derived impregnated with iron oxide biochar for batch and column studies. Sci. Rep. 2024, 14, 18102.

(10) Wu, S.; Zhu, Z.; Si, D.; Zhao, C.; Feng, H.; Zhang, Q.; Wang, J.; Zhou, D.; Wang, Y. Titanium dioxide-loaded biochar composite simultaneously reduces arsenic mobilization and methane emissions in flooded paddy soils. Biochar 2026, 8, 89.

(11) Navarathna, C. M.; Karunanayake, A. G.; Gunatilake, S. R.; Pittman, C. U.; Perez, F.; Mohan, D.; Mlsna, T. Removal of arsenic(III) from water using magnetite precipitated onto Douglas fir biochar. J. Environ. Manage. 2019, 250, 109429.

(12) Smedley, P. L.; Kinniburgh, D. G. A review of the source, behaviour and distribution of arsenic in natural waters. *Appl. Geochem.* 2002, *17*, 517–568.

(13) World Health Organization. *Guidelines for Drinking-Water Quality*, 4th ed.; WHO: Geneva, 2022.
